# Effectiveness of a low-fructose and/or low-sucrose diet in decreasing insulin resistance (DISFRUTE study): study protocol for a randomized controlled trial

**DOI:** 10.1186/s13063-017-2043-z

**Published:** 2017-08-07

**Authors:** Santiago Domínguez Coello, Lourdes Carrillo Fernández, Jesús Gobierno Hernández, Manuel Méndez Abad, Carlos Borges Álamo, José Antonio García Dopico, Armando Aguirre Jaime, Antonio Cabrera de León, the DISFRUTE Group, Nieves Expósito Álvarez, Fátima Fúster Jorge, Juan Carlos Marquez García, Juliana Martín Conde, Laura Moreno Calvo, Juan José Pérez Valencia, Ana Plasencia Barrera, Rafael Valcárcel López, Jorge Conde Pérez, José A. Fernández Hernández, Raquel García Álvarez, Manuel Ramos Fuentes, Concepción Ramos Ramos, Alicia Rodríguez Díaz, Myriam Sánchez Pérez, María J. Brito Cabrera, Mercedes Dorta Espiñero, Oscar Ginovés Sierra, Ana González Licerán, Javier Lorenzo Toledo, Rosa A. Marrero Delgado, Eva M. Morales Medina, Nayra Negrín Torres, Eduardo Orihuela Cabrera, Betty Pérez González, Oscar Guillermo Pérez Martín, Andrés Ramos Hernández, Soraya Sotto Rodríguez, Gilberto Gorrín Vargas, Candelaria Correa Hernández, Maite Díez Martín, Beatriz García Sanchez-Real, Alicia A. López Díaz, Francisco Rodríguez López, María C. Rodríguez Santos, María Candelaria García Méndez, Daisy Herrera Hernández, José. R. Hernández Lorenzo, José Martín Hernández, Antonio M. Reyes Rodríguez, José M. Rodríguez Hage, Inmaculada Sanz Sánchez, Luisa María Fernández Palacio, Piedad González Torrado, Tomás Higueras Linares, Alberto Martel Martell, Marta Pérez Souto, Nélida Olivares Hernández, Teresa Valdés Bilbao, Olga T. Gómez López, Ubaldo González Rodríguez, Mariana Morales López, María Calviño Oliver, Sara Farrais Villalba, Ascención C. García Morín, María D. Marrero Díaz, José A. Milena Jiménez, María P. Morales García, María V. Muñoz Fernández, Dulce E. Carmona Piñero, María R. García Marrero, Genoveva Pérez Pérez, Eduardo Puerta del Castillo, Juan L. Delgado Estévez, María T. Diestre Ortín, Carlos J. Fernández Barreto, María J. Pérez Hernández, Carmen Fuertes Valencia, Almudena Hernández Vázquez, Carmen Jiménez Velado, Carmen Afonso Navarro, Ana D. Dorta León, Blanca González González, Jose M. Afonso Luis, María J. García Roger, Antonia M. Pérez Cejas, Luz M. Pérez Hernández, José M. Salamanca Hernández, Rosa M. Suárez Díaz, Miguel Pico Picos, Dácil Díaz Gómez

**Affiliations:** 1 Unidad de Investigación de la Gerencia de Atención Primaria de Tenerife y del Hospital Universitario Nuestra Señora de la Candelaria, Santa Cruz de Tenerife, Canary Islands Spain; 2 Laboratorio de Análisis Clínicos, Hospital Universitario de Canarias, La Laguna, Canary Islands Spain; 3 Área de Medicina Preventiva y Salud Pública, Universidad de La Laguna, San Cristóbal de La Laguna, Canary Islands Spain; 4 Centro de Salud La Victoria de Acentejo, C./Domingo Salazar 21, 38380 La Victoria de Acentejo Santa Cruz de Tenerife, Canary Islands Spain

**Keywords:** Insulin resistance, Fructose, Sucrose, Clinical trial, Primary care

## Abstract

**Background:**

Research published to date on the relationship between insulin resistance (IR) and fructose consumption is scarce, has used different methods, and has yielded sometimes contradictory results. This study aims to determine whether a low-fructose and/or low-sucrose diet supervised by a physician or nurse decreases IR compared to a standard diet.

**Methods/design:**

This field trial is located at primary care centers. The participants are adults aged 29 to 66 years, with a Body mass Index (BMI) between 29 and 40.99 kg/m^2^ and without diabetes. To date, 245 participants have been assigned randomly to the low-fructose diet intervention group (LFDI) at health centers in the western health service zone of Tenerife island, and 245 to the standard-diet control group (SDC) at health centers in the eastern health service zone. Recruitment is opportunistic and is carried out by physicians and nurses at participating health centers. Initially (baseline), and after 24 weeks of intervention, dietary records, physical activity, waist circumference, BMI, blood pressure, fasting blood glucose and insulin concentrations (HOMA2-IR) and lipid profile are recorded; blood glucose and insulin and lipid profile are also recorded 2 h after a 75-g glucose overload. After 48 weeks (24 weeks after the intervention), fasting blood samples are again obtained and a physical examination is performed. All tests and measures are repeated and recorded except dietary records, physical activity and oral glucose overload. Low-fructose diets are designed by calculating free and total (free + fructose associated with sucrose) fructose contents in standard diets, and removing foods with a fructose content in the highest quartile for the amounts in the standard diet.

Participants in both groups are prescribed a diet that contains 30 to 40% less than the participant’s energy requirements. The primary endpoint is change in HOMA2-IR between baseline and week 24, and other outcomes are change in HDL-cholesterol, LDL-cholesterol, triglycerides , waist circumference to height ratio and BMI. The secondary endpoint is change in HOMA2-IR between week 24 and week 48 together with the outcomes noted above. Comparisons between groups for variables used to indicate IR levels are done with a Student’s *t* test for unpaired variables or the Mann-Whitney *U* test if the distribution is not normal. Multivariate regression models will be used to control for confounding factors not accounted for in the study design, and for independent prognostic factors.

**Discussion:**

If the dietary intervention being tested, i.e., a diet low in fructose/sucrose, is able to reduce IR, the results – if translated into regular clinical practice – could provide a hitherto unavailable tool to prevent type-2 diabetes mellitus.

**Trial registration:**

ISRCTN, ID: ISRCTN41579277. Registered retrospectively on 15 November 2016.

**Electronic supplementary material:**

The online version of this article (doi:10.1186/s13063-017-2043-z) contains supplementary material, which is available to authorized users.

## Background

The worldwide obesity epidemic currently affecting industrialized countries is causing high rates of morbidity and mortality from related diseases and disorders such as diabetes and cardiovascular disease. The influence of suboptimal dietary habits in the appearance of obesity has led many researchers to study the hypothetical role of fructose. One systematic review failed to find support for an effect of fructose on increases in body weight [1]. However, the short follow-up period for most participants, for a mean of 4 weeks in trials with an isocaloric diet and 1.5 weeks in those with a hypercaloric diet, was a major source of uncertainty according to the authors of this review. Other sources of uncertainty noted were heterogeneity among groups of participants (13 trials involved patients with diabetes, 7 involved patients with overweight or obesity, and 21 involved patients with normal body weight), the low number of participants in many trials (13 included 10 participants or fewer, 12 included 11 to 25, and only 5 included more than 25 participants), and the sensitivity to imputations in crossover trials. Nevertheless, the association between excess lipid storage as manifested by obesity and insulin resistance (IR) has long been recognized [2]. Insulin resistance is a metabolic situation characterized by a decrease in the physiological response of peripheral tissues to the action of insulin for cell nutrition and the maintenance of glucose homeostasis. This indicator is strongly predictive of type-2 diabetes mellitus (DM) and cardiovascular disease [3].

The role of fructose in the development of IR in the liver is controversial. This monosaccharide is present especially in fruit, and has become a widely used additive in many food industry products such as soft drinks and nonalcoholic beverages, generally in the form of fructose-enriched corn syrup [4]. Moreover, most frequently consumed manufactured food products contain sucrose as an additive; this disaccharide consists of equal proportions of fructose and glucose.

Different mechanisms have been proposed to explain the effect of fructose versus glucose consumption on the appearance of IR. One mechanism is related to the liver metabolism of each monosaccharide. Excessive fructose consumption increases de-novo lipogenesis and can lead to dyslipidemia, increased visceral fat, overweight and obesity. Glucose metabolism in the liver may limit the production of substrates characteristic of de-novo lipogenesis since this is regulated by phosphofructokinase which is inhibited by ATP and citrate under conditions of energy intake above the individual’s requirements. However, fructose is metabolized in the liver via fructokinase in a manner independent of energy status, and so lacks the limiting control mechanism that operates for glucose. A consequence is that the consumption of large amounts of fructose does not halt de-novo lipogenesis; this, in turn, can increase the amount of hepatic fat and thus induce IR in the liver [5]. Another proposed mechanism is related to the possible satiating effect of fructose. Consumption of this sugar stimulates insulin secretion which, in turn, leads to leptin release from adipocytes and the inhibition of ghrelin secretion in the gastrointestinal tract. These changes stimulate brain centers that regulate satiety and energy homeostasis. However, fructose consumption does not lead to acute stimulation of insulin secretion which would attenuate the stimulation of leptin secretion and ghrelin inhibition. These effects, in turn, influence the regulatory action of these hormones on energy balance in the central nervous system. In people who consume large amounts of fructose, the lower circulating levels of insulin and leptin together with increased ghrelin concentration may lead to increased calorie intake, thereby contributing to weight gain and obesity when high-fructose diets are consumed chronically [6]. Page and colleagues showed that glucose but not fructose consumption reduced activation of the hypothalamus, insula and striatum – brain regions that regulate appetite, motivation and reward processing [7].

Several recent epidemiological studies have suggested a plausible relationship between fructose consumption and IR. Thus far, interventions have been tested to increase and decrease fructose consumption. In two separate studies Aeberli and colleagues. found that even with moderate levels of fructose or sucrose consumption (40 and 80 g, respectively) compared to moderate glucose consumption (40 g), atherogenic low-density lipoprotein (LDL) particles were increased along with total and LDL-cholesterol, and IR in the liver. However, they found no differences in IR (HOMA2-IR), possibly because of the short duration of the study (3 weeks) [8, 9]. Stanhope and colleagues also found increases in LDL-cholesterol, non-high-density lipoprotein (HDL)-cholesterol, apolipoprotein B, postprandial triglycerides, cholesterol and remnant triglycerides, as well as small dense LDL-cholesterol, in persons who obtained 25% of their energy requirements from beverages sweetened with fructose-enriched corn syrup compared to persons who consumed a glucose-rich diet [10]. Maersk and colleagues studied 17 men and 30 women with overweight for a 6-month period to compare the influence of a sucrose-rich beverage (20% of total energy requirements) with three isocaloric diets (one with low-fat milk, one with aspartate as a beverage sweetener, and one with water). They found increases in visceral fat, liver and muscle triglycerides and elevated fasting plasma triglyceride levels. Interestingly, these effects were not accompanied by changes in body weight [11].

Most trials designed to reduce fructose consumption have involved children and adolescents as frequent consumers of sugar-rich beverages. A controlled double-blind trial that enrolled children showed that a dietary education programs designed to reduce sugar and fructose consumption by substituting low-calorie sweetened beverages for sugar-containing beverages had beneficial effects on Body Mass Index (BMI) [12]. A randomized trial that followed with overweight and obesity for 2 years found that the increase in BMI was smaller in participants who consumed fewer sugar-sweetened beverages [13]. Neither of these trials investigated IR, and calorie intakes in the intervention group were lower than in the control group. Another trial with African-American and Latino adolescents found that reducing the consumption of added sugar improved insulin sensitivity even when body weight or body fat was not reduced [14]. In that study, calorie intake in terms of kcal/day was again higher in the control group than in the two intervention groups (nutrition education, and nutrition education plus strength training). The only study in adults that we are aware of was designed to analyze the effect of decreased fructose consumption on body weight and IR [15]. This study compared two isocaloric diets with no added sugars but containing different amounts of fructose (≤20 g versus 50–70 g). The decrease in BMI was greater in the latter group, and IR (HOMA) was also lower although the difference between groups was not statistically significant (*p* = 0.12).

In light of the gaps in our current knowledge, we designed a field trial to test the effects of decreased fructose consumption on IR under real-life conditions in participants who consume an isocaloric diet.

We describe here a single-blind, multicentre, randomized controlled field trial lasting 24 weeks that is being carried out at primary health care centers, designated the DISFRUTE (DISminución de la FRUctosa en TEnerife, i.e., Decrease in fructose in Tenerife) study. The aim of the trial is to determine whether a decrease in dietary free or sucrose-associated fructose is effective in decreasing IR.

## Methods/design

The *primary objective* of this study is to determine whether decreasing the consumption of foods rich in fructose or sucrose leads to a decrease in IR after 24 weeks in a population with obesity, independent of a reduction in calorie intake.

The *secondary objective* is to determine whether after 48 weeks (24 weeks after the end of the intervention according to protocol), IR levels remain unchanged compared to the end of the 24-week intervention period.

### Design

A single-blind, multicenter, randomized controlled **f**ield trial with two groups of participants (low fructose/sucrose diet versus standard diet) recruited at primary care health centers in Tenerife island (Canary Islands, Spain). Sample size: because of the lack of consensus in the academic literature regarding the cutoff value of HOMA2-IR that defines the presence of IR, we opted to use body weight change. We estimated a mean weight loss of 4 kg (1.43 kg/m^2^ BMI assuming a mean height of 1.67 m for the Canary Islands’ population [16]), a significance level of *p* ≤ 0.05, 80% power and a 20% dropout/loss to follow-up rate. A weight loss of 4 kg was estimated based on maximum weight loss in the only interventional trial reported to date of the effect of lower fructose consumption on body weight and IR [15]. Dietary intervention trials aimed at studying weight loss have reported similar amounts of weight loss after 6 months [17]. In the trial reported here, in which both groups consume an isocaloric diet, we do not anticipate differences between the groups in the amount of weight lost. Based on these calculations our estimated sample size for each of the two groups is 245 participants.

The primary outcome measure is insulin resistance (IR) which is estimated from fasting serum glucose and insulin concentrations with the computer-based Homeostasis Model Assessment system (HOMA2-IR) at baseline, 24 and 48 weeks. The secondary outcomes are BMI, waist circumference (waist to height ratio) and blood pressure which are measured at baseline, 4, 8, 12, 16, 20, 24 and 48 weeks, and total cholesterol, HDL-cholesterol, LDL-cholesterol and triglycerides which are measured at baseline, 24 and 48 weeks.

### Study participants

Four hundred and ninety patients who participate voluntarily; our intention is to include at least 40% men.

#### Inclusion criteria

Adults, aged between 29 and 66 years, BMI between 29 and 40.99 kg/m^2^.

#### Exclusion criteria

Pregnancy (women), behavioral eating disorders, relevant gastrointestinal disease (ulcerative colitis, Crohn’s disease, celiac disease, digestive tract cancer), excessive alcohol consumption (>28 U or 280 g/week in men, >17 U or 168 g/week in women), cardiovascular event in the last 3 months or unstable cardiovascular disease, diabetes, polycystic ovary disease, treatment with any medication that could alter insulin sensitivity or body weight (corticosteroids, antipsychotics, antidepressants), pharmacological treatment for clinical or subclinical hypothyroidism, hyperthyroidism, depression, psychosis, microalbumin/creatinine ratio >100 mg/g or stage-IIIB or higher chronic kidney disease (glomerular filtration rate <45 mL/min), use of medication requiring frequent dose adjustments and low intellectual or mental functioning that could interfere with the participant’s compliance with the recommendations. If the result of the glucose overload test is blood glucose ≥200 mg/dL the participant is excluded if the physician opts to add medication or insulin to the dietary and physical exercise regime.

### Randomization

Participants are randomized by health care zone. Patients in the low-fructose diet intervention group (LFDI) are assigned to health centers in the western zone of Tenerife island, and patients in the Canary Islands Health Service standard-diet control (SDC) group are assigned to the eastern zone of the island. This randomization system is used to prevent contamination bias. If patients randomized to both groups attended the same health center this source of bias would be difficult to avoid because many patients in the LFDI group are likely to have relatives or friends in the SDC group, and vice versa. Contamination could also occur between staff members at a given health center. Sixteen primary health centers are participating in this study; all of them belong to the same health care administration district (Gerencia de Atención Primaria de Tenerife). Given that the total area of the island of Tenerife is relatively small, the characteristics of the population served by all health centers are assumed to be similar. The longest distance between participating health centers is 64 km. All centers and staff provide the same suite of services. The variations between centers in the catchment population assigned and the numbers of patients under each staff physician’s and nurse’s care are similar. We therefore do not anticipate between-group differences in the baseline data or systematic biases in the results of the care provided at different centers.

### Recruitment

Physicians and nurses recruited participants with an opportunistic method. To facilitate recruitment we designed a digital application for electronic medical records that make it possible to identify patients who could hypothetically be recruited on the basis of our inclusion and exclusion criteria. The first screen of the electronic medical record displays an alert indicating that the patient is potentially eligible for inclusion. The nurse or physician explains the nature and aims of the study to the patient, and if the patient expresses interest in taking part, the physician asks three questions about their motivation. If the patient answers “yes” to all three questions the nurse or physician asks them to sign the Informed Consent Form (see Additional file 1), and then gives the patient a brief Information Pack that includes four pages of a food diary in which the patient is asked to record all foods and beverages consumed each day during a 4-day period that includes one weekend day or holiday. The Information Pack also includes a sheet with instructions on how to record foods and beverages in the food diary (see Additional file 2). On the day that the patient signs the Informed Consent Form, an appointment is scheduled to review the food diary and complete the Case Record Form (CRF). At this appointment the physician or nurse carefully reviews all four daily food diary sheets with each participant to ensure accuracy regarding the types, sizes and portions of each food and beverage. During this appointment the participant is interviewed about their physical activity and the information provided is entered by the physician or nurse into the Minnesota Leisure-time Physical Activity Questionnaire form. Body weight, height and waist circumference are recorded, and blood pressure is measured twice and recorded as the average of the two values. At the end of this appointment the participants is given an appointment for a blood test (week 0). The blood sample is provided after an overnight fast, and is used to measure plasma glucose, insulin and lipid concentrations (total, LDL- and HDL-cholesterol and triglycerides). At week 0 we also record thyroid stimulating hormone (TSH), total thyroxine (T_4_) if TSH is altered, and urinary microalbumin/creatinine ratio (to rule out relevant thyroid or kidney disease). All participants undergo an oral glucose overload test (75 g) to record blood insulin, glucose and lipids 2 h later. All participating health center staff are reminded to use the same equipment in each appointment to record blood pressure, weight and height. All devices are calibrated regularly by the Canary Islands Health Service. Nonstretchable measuring tape is used to record waist circumference by situating the tape halfway between the lower margin of the last rib and the iliac crest with the participant breathing normally.

### Intervention

All participants sign an Informed Consent Form. The study was approved by the Ethics Committee of Nuestra Señora de La Candelaria University Hospital. The intervention (except for nutrition counseling), number of appointments and laboratory tests are the same in both groups.

Participants in the LFDI group are advised to eat a low-fructose diet designed on the basis of standard diets (1000, 1250, 1500, 1750, 2000, 2250, 2500 or 2750 kcal/day) recommended by the Canary Islands Health Service [18]. Low-fructose diets are designed by calculating free and total (free + fructose associated with sucrose) fructose contents in standard diets. Foods with a fructose content in the highest quartile for the amounts corresponding to the standard diet are removed from the standard diet. The format and graphical appearance of the information about the participant’s diet are identical in both groups. Physicians and nurses who prescribe the standard diets are unaware of which foods have been excluded from the low-fructose diets. To avoid contamination bias while recruitment is currently underway, the present report does not include information on the composition of the low-fructose diets.

Nutrition counseling and reinforcement take place at weeks 2, 4, 8, 12, 16 and 20. During this process we emphasize to participants the importance of involving in the intervention the person in the household who purchases and/or prepares food. At week 2, after the physician or nurse has reviewed the nutritional information, intervention via individualized nutrition counseling begins. At week 24 a second blood sample is obtained for analysis. Two weeks before week 24 the participants are given a new Information Pack containing the same materials as the initial Information Pack at the start of the study, with instructions on how to record foods and beverages consumed during 4 days (including one weekend day or holiday). On the day of the appointment to provide a new blood sample, the participant meets first with the physician or nurse to review their food diary in detail.

**Fig. 1:**
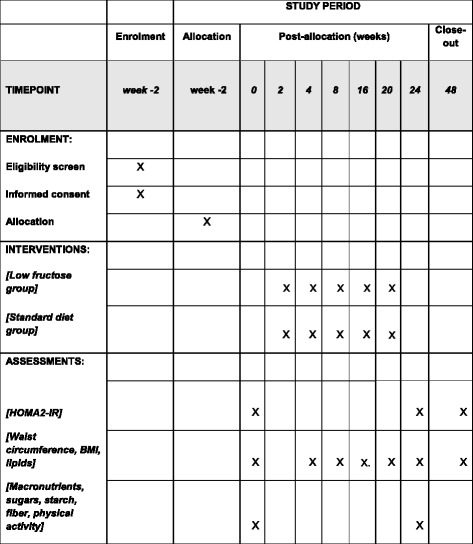
Schedule of enrollment, interventions and assessments

**Fig. 2:**
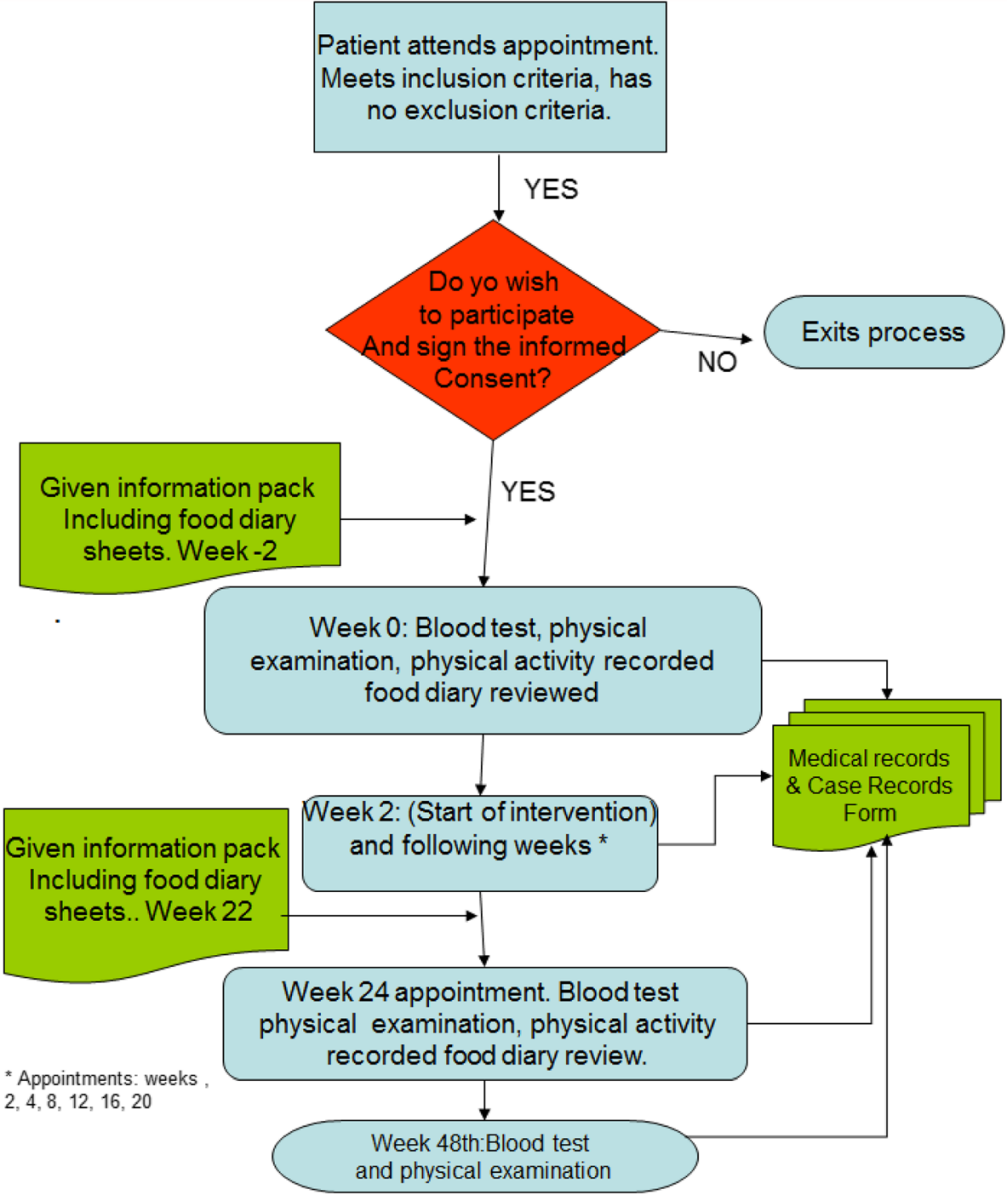
Patient recruitment and follow-up process

The schedule of enrollment, interventions and assessments, which is based on the Standard Protocol Items: Recommendations for Interventional Trials (SPIRIT) figure, is shown in Fig. 1 and the process for participant recruitment and follow-up is shown in Fig. 2.

#### Physical activity and dietary intervention in both groups

To determine leisure time physical activity we use the Minnesota Questionnaire which has been validated for the Spanish population [19, 20]. Participants are also asked about their physical activity at work. Physical activity is recorded for the week prior to the appointment and for the six previous months. If the participant reports physical activity equivalent to moderate or brisk walking for 150 min per week or more, they are encouraged to continue this level of activity. If less physical activity is reported, the participant in encouraged to devote 150 min per week to brisk walking.

When the research team receives the four daily food diaries and information on physical activity from the physician or nurse, team members analyze the information to make general dietary recommendations, and advise the health center staff member on the kcal/day the participant’s diet should contain, and on the type and amount of physical exercise to prescribe. To evaluate adherence to the prescribed diet, the physician or nurse is reminded to advise participants to record a 24-h recall of foods and beverages consumed at weeks 8, 12, 16 and 20. The kcal/day in the prescribed diet are calculated as 30 or 40% less than the kcal/day of the participant’s energy requirements for their ideal weight according to age, sex and physical activity. Ideal weight is calculated as the product of 25 multiplied by the square of height (measured in meters); physical activity is evaluated from information provided in the Minnesota Questionnaire, and participants are asked specifically about the nature of their employment. Members of the research team classify physical activity into four levels of intensity: mild, moderate, vigorous or very vigorous. Energy requirements in kcal/kg/day are calculated with the protocol for persons with obesity used by the Canary Islands Health Service (based on recommendations of Spanish Ministry of Health Guideline, 2008 [21]). For men, the requirements are assumed to be 42, 46, 54 and 62 kcal/kg/day, respectively, for each of the four levels of intensity, and for women the requirements are assumed to be 36, 40, 47 and 55 kcal/kg/day. These figures for energy requirements are reduced by 5% for participants 45 to 49 years old, by 10% for those 50 to 59 years old, and by 20% for those aged 60 years or older. After energy intake equivalent to 30 to 40% less than the individual’s energy requirements is calculated, the participant is advised to consume the diet that most closely approximates this figure (1000, 1250, 1500, 1750, 2000, 2250, 2500 or 2750 kcal/day). Additional file 3 provides an example of the standard 1750 kcal/day diet, how the energy requirements for the recommended diet are calculated and one example of the information provided by the researchers to participating physicians and nurses.

On the basis of the available data for the Canary Islands’ population, we decided that the energy intake restriction should ideally represent approximately 35% of each participant’s energy requirements. Mean energy intake in the general population of the Canary Islands is 2300 kcal/day [22]. The recommended diets for participants with obesity provide 1250 to 1500 kcal/day for women (mean 1350 kcal/day) and 1500 to 1800 kcal/day for men (mean 1650 kcal/day) [23]. The mean energy intake for men and women is, therefore, 1500 kcal/day. The difference between the mean energy intake of 2300 kcal/day in the Canary Islands’ population and the mean energy intake of 1500 kcal/day is 800 kcal/day, i.e., 35% of the daily energy consumption. This calculation is in agreement with the energy restriction of the 30–40% recommended by the above mentioned guideline [21].

Although we decided on a range of energy intake restrictions between 30 and 40% to match the prescribed diets as closely as possible to the individual requirements we calculated, in practice the restriction in the prescribed diets closely approximates 35% of the participants’ energy requirements.

### Data recording and analysis

A specifically designed Case Record Form (CRF) is used to record personal contact information, socioeconomic class, personal and family antecedents, and to compile data for 24-h recall food consumption and physical activity. The CRF is shown in Additional file 4. Socioeconomic class is stratified as low, middle and high according to the ICE method Incomes, Crowding index, Education [24] which has been weighted and validated for the Canary Islands’ population. Mean nutritional value of each of the four daily records is used as the baseline value (week 0), and is estimated mainly from the Mataix Spanish food composition tables for macronutrient, sugar and calorie intakes [25]. Blood samples are analyzed at the Canary Islands University Hospital Complex laboratory. Insulin is measured with a chemiluminescence immunoassay method and an Architect i2000SR immunoassay analyzer (Abbott Diagnostics). Glucose, total and HDL-cholesterol and triglycerides are measured with enzymatic methods (Cobas c602, Roche Diagnostics). Microalbumin/creatinine ratio is determined with an immunoturbidimetric method (Cobas c501, Roche Diagnostics). To measure TSH and T_4_ a chemiluminescence immunoassay is used with a Cobas e602 processor (Roche Diagnostics). The value for LDL-cholesterol is obtained with the Friedewald formula if triglycerides are below 400 mg/dL. Insulin resistance is estimated from fasting serum glucose and insulin concentrations with a computer-based Homeostasis Model Assessment system (HOMA2-IR) provided by the Oxford Centre for Diabetes Endocrinology and Metabolism [26] (http://www.dtu.ox.ac.uk/).

To normalize the methods used for measurements, data recording and intervention the research team met with all physicians and nurses who collaborate in the study 2 months before field work began. During a 7-h session team members explained the methodology and instructed the health center staff members in how to record data from the food diaries and how to implement the dietary interventions. All collaborating health center staff members were given a manual with photographs illustrating food item sizes, portion sizes and weights in order to minimize recording biases, and they were also given graphical material illustrating how to estimate the volume of different beverages. One year after the start of the recruitment a second meeting was held with all collaborating health center staff members to update them on the progress and results of the study thus far.

Data recording is handled by an externally contracted data manager whose fees are covered from funding for this study. Data analysis will be performed by the researchers, and no member of the entity responsible for fund management will participate in data recording or data analysis. No member of the entity responsible for fund management participated in the drafting, review or revision of this manuscript.

CRFs and food diaries are emailed to an account which is accessible only by three of the principal investigators and the data manager. All records are printed to facilitate study and are kept in a locked cabinet. The results of all laboratory analyses are sent in encrypted form by the laboratory head to the principal investigator, who then sends them personally to the individual physicians responsible for each participating patient at each health care center.

The data manager is specifically trained in transcribing the data from CRFs, laboratory reports and food diaries to the database used for statistical analyses (Statistical Package for the Social Sciences; see below under “Statistical analysis”). The data from CRFs, laboratory reports and food diaries are not exported digitally to the statistical software for two reasons. Firstly, funding limitations meant that the study could not commence until additional funding was obtained to cover the basic costs of the study. Secondly, the timeline for this study necessitates food diaries to be obtained and recorded (four during week 0 and four at week 24), and data entry from the diaries requires interpretation by the data manager, who is specifically trained to interpret the different types of foods and to use the correct unit of measurement for different food items (mass, volume, portion, etc.). However, every 15 days the accuracy of data entry was verified by checking all data entered in each of the databases against the original data sources.

### Monitoring

The research team is responsible for monitoring the study. At week 24 the participants are asked whether they had side effects from the prescribed diet (CRF). At the time of writing the only side effect reported by any participant was constipation. The research team is also responsible for systematically reviewing the causes of withdrawal or dropout. Because no experimental medications or foods are used in this study, we believe that there is no need to create a specific data monitoring committee made up of professionals who are not involved in the research per se.

### Statistical analysis

Before-after changes in IR, laboratory indicators and anthropometric values are determined with Student’s *t* test for paired samples. Comparisons between the intervention and control groups for variables indicative of IR are done with Student’s *t* test for independent samples when the frequency distribution is normal, or with the Mann-Whitney *U* test when the distribution is not normal. For these comparisons multiple linear or regression models are used as appropriate to detect possible confounders or independent prognostic factors for IR that are not controlled for in the trial design. Student’s *t* test for paired samples will be used to analyze changes between week 0 and week 24 in the main outcome variables in each group (HOMA2-IR, fasting blood glucose and insulin, and these same measures after an oral glucose overload) and the secondary outcomes (fasting LDL-cholesterol, HDL-cholesterol and triglycerides, and these same measures after a glucose overload; waist circumference, waist to height ratio, BMI and blood pressure). To verify compliance with the recommended diets the same analysis will be done for total calorie intake and individual nutrient intakes. These analyses will record results for the variables noted above between week 24 and week 48, and between week 0 and week 48. Student’s *t* test for independent samples will be used to analyze the differences between groups observed from week 0 to week 24 for the variables noted above. As potential confounders we will consider calorie intake, variations in nutrient intakes other than fructose, and physical activity. If differences are detected between groups at the start of the study in HOMA2-IR, any nutrient intake or physical activity, appropriate adjustment will be used in the outcomes analysis. An intention-to-treat analysis will be done taking into account losses to follow-up, and using the most recent changes and measurements available for participants who did not complete the study. All hypothesis tests will be two-sided with a significance level of *p* < 0.05, and all statistical analyses will be done with the NT Professional v. 21.0 (IBM) statistical package (SPSS) for PCs in a Windows environment. More specific information about the trial and protocol is summarized in the Standard Protocol Items: Recommendations for Interventional Trials (SPIRIT) Checklist (Additional file 5).

## Discussion

The aim of this study is to evaluate the effectiveness of a low-fructose/sucrose diet compared to a standard diet in lowering IR in obese patients without DM after an intervention period of 24 weeks. Overall reductions in fructose/sucrose consumption are evaluated with no distinction between added sweeteners and sugar naturally present in foods. A diet low in foods with natural fructose content is prescribed for the intervention group, but participants in both groups are instructed to refrain from consuming foods with added sugar. This approach is used because it reflects the usual care offered at participating health centers, and in an effort to ensure comparable food intakes in kcal/day in both groups. To evaluate the difference in fructose intake our reference is the mean value from four daily food diary records from the end of the intervention period (week 24) compared to four daily food diary records at week 0. Although the evidence at present is insufficient to claim that four food diaries are sufficient to provide a reliable analysis of fructose or sucrose intakes, most studies that have estimated these intakes to date have used 24-h recall or (usually four) food diaries [27].

At present, the amount of fructose intake that could be harmful to health is unknown. A trial such as this one, carried out in the primary care setting, should provide valuable information for future efforts to control IR. Because the restricted calorie diet prescribed for our participants is similar in both groups, we expect to see similar weight reductions in both. Our hypothesis is that under our trial conditions, the reduction in IR will be greater in the group with the low-fructose diet than in the SDC. Accordingly, if the dietary intervention being tested, i.e., a diet low in fructose/sucrose, is able to reduce IR, the results – if translated into regular clinical practice – could provide a hitherto unavailable tool to prevent type-2 DM.

A potential limitation of this type of study is the likelihood that many participants will under-report some of their food intakes, as has been shown for persons with obesity. We will analyze the energy intake:basal metabolic rate ratio with a cutoff value of 1.16 for women and 1.19 for men to determine whether participants are under-reporting any food intakes [28].

Another limitation of this study is that processed food labels generally do not list the amounts of added fructose and sucrose, although most products indicate (in grams) the amounts of mono- and disaccharides as part of the total carbohydrate content. We are, therefore, using available national and international databases to determine the amounts of fructose consumed. If no information on fructose content is available in these sources, we calculate nutrient contents from the recipe used to prepare certain traditional dishes. Our sources of macronutrients and sugars are detailed in Additional file 6 (in Spanish) and Additional file 7 (in English). The term “sugars” on a product label is assumed to refer to total sucrose content only if no information on the content of each type of sugar is available from the databases, or if the sugar content cannot be estimated from the recipe used to prepare a given dish at home.

### Trial status

This trial is currently ongoing. Recruitment began in May 2014.

## Additional files

Additional file 1: Consent Form. (PDF 27 kb)

Additional file 2: Food diary and instructions for completion. (DOCX 31 kb)

Additional file 3: 1750 kcal/day diet and example of a prescribed diet. (PDF 448 kb)

Additional file 4: Case Record Form. (DOCX 74 kb)

Additional file 5: SPIRIT 2013 Checklist: recommended items to address in a clinical trial protocol and related documents. (DOCX 54 kb)

Additional file 6: Foods included to date and sources of macronutrients and sugars (Spanish). (DOCX 35 kb)

Additional file 7: Foods included to date and sources of macronutrients and sugars (English). (DOCX 40 kb)

## Abbreviations

DISFRUTE: DISminución de la FRUctosa en TEnerife
BMI: Body Mass Index
LFDI: Low-fructose diet intervention group
SDC: Standard-diet control group
HOMA: Homeostasis Model Assessment
DM: Diabetes mellitus
LDL: Low-density lipoprotein
HDL: High-density lipoprotein
CRF: Case Record Form
TSH: Thyroid stimulating hormone
ICE: Incomes, Crowding Index, Education
