# Supplementary material for: Effectiveness of a low-fructose and/or low-sucrose diet in decreasing insulin resistance (DISFRUTE study): study protocol for a randomized controlled trial
Source: Trials. 2017 Aug 7;18:369. doi: 10.1186/s13063-017-2043-z (PMC5547514; doi:10.1186/s13063-017-2043-z)
Supplement: Supplementary file 1 — Consent Form. (PDF 27 kb) [file 13063_2017_2043_MOESM1_ESM.pdf]

CONSENTIMIENTO INFORMADO PARA PARTICIPACIÓN EN UN ESTUDIO DE INVESTIGACIÓN

Yo, ____DNI:____________

Acepto participar en el estudio de investigación denominado DISFRUTE (expediente Instituto Carlos III, PI12/00231) que me propone mi médico o mi enfermero acerca de una intervención dietética. En este estudio se le dará consejo a usted y a otras personas sobre una dieta para que bajen de peso. A otro grupo de pacientes se le recomendará una dieta diferente. El objetivo es comparar qué dieta consigue mejores resultados.

El investigador principal es el Dr. Santiago Domínguez Coello y trabaja en el Centro de Salud de La Victoria de Acentejo y en la Unidad de Investigación del Hospital Universitario Ntra. Sra de La Candelaria. Este proyecto ha sido aprobado por el **comité de ética** del Hospital y está financiado con **fondos públicos** provenientes del gobierno de España.

Los investigadores **me han informado** de los objetivos de su trabajo y sé que **mi participación es voluntaria** y que puedo retirarme de este estudio en cualquier momento, sin que ello afecte a mis derechos de ninguna manera. En cuanto a los **datos personales** que facilito para este estudio, entiendo que serán **tratados y custodiados con respeto a mi intimidad y anonimato**, según la vigente normativa de protección de datos (Ley 15/1999 de 13 de diciembre). También sé que tengo derecho a de acceder, rectificar y cancelar mis datos mediante solicitud ante el investigador principal (Dr Domínguez) en la dirección de contacto que figura en este documento.

Doy mi consentimiento a los investigadores de este estudio para que me realicen las extracciones de sangre que están protocolizadas para su análisis, donde se medirán la glucosa, la insulina, el colesterol total, hdl colesterol, ldl colesterol y triglicéridos. Asimismo**, acepto dar la información** sobre mi edad, clase social, peso, talla, cintura abdominal, tensión arterial y sobre antecedentes personales y familiares de diabetes, y autorizo a los investigadores del estudio a consultar en mi historia clínica aquellos datos relacionados con el estudio.

Declaro que he leído el presente documento, me lo han explicado y comprendo lo que dice. Y firmo este consentimiento de forma voluntaria para participar en este estudio mientras yo no decida lo contrario. Al firmar este consentimiento no renuncio a ninguno de mis derechos. Recibiré una copia de este documento para guardarlo y poder consultarlo en el futuro.

Firma del participante: Firma del investigador:

Fecha:

(I.P: Santiago Domínguez Coello. Unidad de Investigación HUNSC. Tfno: 922600602.)
