# Supplementary material for: Effectiveness of a low-fructose and/or low-sucrose diet in decreasing insulin resistance (DISFRUTE study): study protocol for a randomized controlled trial
Source: Trials. 2017 Aug 7;18:369. doi: 10.1186/s13063-017-2043-z (PMC5547514; doi:10.1186/s13063-017-2043-z)
Supplement: Supplementary file 2 — Food diary and instructions for completion. (DOCX 31 kb) [file 13063_2017_2043_MOESM2_ESM.docx]

**ESTUDIO DISFRUTE**

(Inicio)

Estimado participante en este estudio:

En primer lugar queremos decirle que todos los registros de los alimentos que usted va a anotar a continuación serán tratados con estricta confidencialidad. Su contenido será volcado en una base de datos por un equipo de profesionales quienes, una vez analizado, remitirán los resultados a su médico y/o su enfermero para que éstos realicen la intervención alimenticia que se le ha asignado.

Le pedimos que si tiene cualquier duda o problema se comunique con nosotros de lunes a viernes en horario laboral al teléfono 922581900 /01, donde se le dará la información que precise. Si el personal responsable no le puede atender en ese momento o está ausente, le pediremos que deje su nombre y teléfono de contacto para comunicarnos con usted a la mayor brevedad. Si quiere comunicarse vía correo electrónico, hágalo a estudiodisfrute@gmail.com

Queremos recordarle que este trabajo de investigación va dirigido a comparar dos dietas diferentes para bajar de peso. Por ello, independientemente del grupo en el que esté usted incluido el objetivo a alcanzar para beneficio de su salud es el mismo: disminuir su peso.

Una vez que haya apuntado usted el registro de cuatro días distintos (uno correspondiente a un sábado o domingo o día no laborable si usted trabaja el fin de semana), le pedimos que los introduzca en el sobre que le adjuntamos y los lleve el día que ha sido citado con su médico o enfermero en el Centro de Salud. Recuerde que antes de proceder a la extracción de sangre los cuatro registros deben haber sido anotados, entregados y valorados por el médico o enfermero.

**MUCHAS GRACIAS POR SU VALIOSA CONTRIBUCION A ESTE ESTUDIO DE INVESTIGACIÓN**.

Por favor, anote todas las comidas y bebidas consumidas durante las 24 horas, incluyendo snacks, azúcar que usted añada, salsas, extras como el aliño de las ensaladas, leche condensada en los cortados y cafés….

Por favor, aunque su médico o enfermero le ayudarán cuando le entregue estos registros, le rogamos que intente ser tan preciso como le sea posible, por ejemplo:

1.- Anote las cantidades y las raciones que come y que bebe: por ejemplo: una taza, una loncha, cucharadas soperas, o de postre, etc. Si se trata de bebidas: una lata, una botella, una cuarta, un vaso…

2.-Anote el tipo de comida, por ejemplo, leche entera o desnatada, vino tinto o blanco, refrescos light o normales, zumos de frutas (si son naturales o envasados)….

3.-Método de cocinado y presentación (papas fritas o arrugadas, fruta natural o envasada, atún natural o en lata, pescado frito, a la espalda, guisado….)

4.-Por favor, indique si el alimento es fresco, congelado, enlatado…

5.- Por favor, escriba las marcas si las conoce: Ejemplos: “cereales de kellogs, de hacendado, de auchan”, “galletas marca gullon, digestive….,

CENTRO: CIP: Profesional: nº:

Fecha:

**NOMBRE:**

**fee**

**Desayuno**

**Media**

**mañana**

**Almuerzo**

**Cena**

**Antes de**

**Acostarse**

**Otras**

**Comidas**

**entre horas**

**Merienda**

**NOMBRE: Fecha:**

**fee**

**Desayuno**

**Media**

**mañana**

**Almuerzo**

**Cena**

**Antes de**

**Acostarse**

**Otras**

**Comidas**

**entre horas**

**Merienda**

**NOMBRE: Fecha:**

**fee**

**Desayuno**

**Media**

**mañana**

**Almuerzo**

**Cena**

**Antes de**

**Acostarse**

**Otras**

**Comidas**

**entre horas**

**Merienda**

**NOMBRE: Fecha**

**fee**

**Desayuno**

**Media**

**mañana**

**Almuerzo**

**Cena**

**Antes de**

**Acostarse**

**Otras**

**Comidas**

**entre horas**

**Merienda**
