# Supplementary material for: Effectiveness of a low-fructose and/or low-sucrose diet in decreasing insulin resistance (DISFRUTE study): study protocol for a randomized controlled trial
Source: Trials. 2017 Aug 7;18:369. doi: 10.1186/s13063-017-2043-z (PMC5547514; doi:10.1186/s13063-017-2043-z)
Supplement: Supplementary file 3 — 1750 kcal/day diet and example of a prescribed diet. (PDF 448 kb) [file 13063_2017_2043_MOESM3_ESM.pdf]

DISFRUTE STUDY

ERDF European Regional Development Fund

FEDER
Fondo Europeo de Desarrollo Regional

1750 KCAL DIET

Food weights refer to raw, cleaned foods (peeled, cored, deboned, skinned, etc.).
Use, as far as possible, measures used at home:

- One dessert spoon is the equivalent of 5 g raw food.
- One tablespoon is the equivalent of 10 g raw food.
- One coffee cup is equivalent to 100 g raw food.
- One ladle is equivalent to 125 g raw food.
- One cup (water glass) is equivalent to 200 mL.

Daily intake of vegetable oils: 30 cm³ (3 tablespoons of olive, sunflower or corn oil).

**BREAKFAST**	Skim milk (200 cm³), alone or with coffee, or 2 servings of low-fat yogurt or 35 g Burgos (cured) cheese or 100 g low-fat cheese.<br>**Also add**: bread or cereals (40 g) or 30 g gofio (wheat or corn flour) and 20 g high-protein food: tuna without oil, cheese, or lean cured meat or sausage.
**MID-MORNING**	50 g white or whole-grain bread<br>20 g high-protein food: tuna without oil, cheese, or lean cured meat or sausage.
**LUNCH**<br>**First course**	⇒**Choose one serving of vegetables** from the following:<br>300 g (raw, clean weight) type A: chard, celery, eggplant, broccoli, squash, mushrooms, cabbage, escarole, curly endive, asparagus, spinach, lettuce, cucumber, bell pepper, radish, tomato,<br>Or 200 g (raw weight) type B: watercress, spring onion, green or string beans, zucchini squash,<br>or round zucchini squash), turnip or leeks.<br>Or 100 g (raw weight) type C: artichokes, onion, Brussels sprouts, beets or carrots.<br>⇒**Also choose one serving of** the following **starchy foods:** 120 g type A: peas or raw fava beans, or 100 g type B: potato stew, potatoes, sweet potatoes or 40 g type C: chick peas, peas, fava beans, green or string beans, lentils, or white or whole-grain bread. Or 30 g type D: rice, pasta, toasted bread, packaged dried mashed potatoes.<br>20 g white or whole-grain bread.
**Second course**	⇒**Choose one serving of the following high-protein foods**:<br>130 g fish or 100 g lean chicken (skin removed), turkey breast, rabbit, veal or beef.
**Dessert**	⇒**Fruit**: 300 g group A (watermelon, grapefruit, melon or papaya), or 150 g group B (oranges, tangerines, plums, kiwis, fresh pineapple, strawberries or apricots),<br>or 120 g group C (apples, pears, peaches, mangos, prickly pears,<br>or 75 g group D (bananas, grapes, cherries, nectarines, medlars or figs).
**MID-AFTERNOON**	50 g white or whole-grain bread<br>20 g high-protein food: tuna without oil, cheese, or lean cured meat or sausage.
**SUPPER**	**Vegetables**: 300 g type A or 200 g type B or 100 g type C.<br>**High-protein foods**: 100 g lean meat or poultry, or 130 g fish or 2 eggs.<br>**Starchy foods**: 120 g type A, or 100 g type B, 40 g type C or 30 g type D.<br>20 g white or whole-grain bread.<br>**Fruit:** 300 g type A, 150 g type B 120 g type C or 75 g type D
**Before bedtime**	One glass of skim milk (200 cm³)

EXAMPLE OF DIETARY RECOMMENDATIONS PROVIDED BY THE RESEARCH TEAM TO PARTICIPANTS' PHYSICIANS AND NURSES

Name of participant

Age: 53 years. Sex: Male. Weight (kg): 93.5. Height (cm): 175.0. BMI (kg/m^2): 30.53
Ideal weight (kg): 76.56
Energy requirements (ER): 76.56 × 46 – age-based reduction (10%): 3128
Recommended diet: (30% to 40% below ER): 1876 to 2189 kcal/day

We recommend for this patient a 2000 kcal/day diet.
On reviewing his food diaries we observe some instances of noncompliance and excess calorie intake.
Firstly, the patient adds sugar to his coffee or barraquito [coffee, fresh and condensed milk, lemon, cinnamon and liqueur]. Instead of sugar, sweetener or saccharine could be used. We note that this participant consumes a large number of servings of yogurt, with a resultant overload of added sugar. We advise him to consume low-fat, unsweetened (0% sugar) yogurt instead, and if it is too bland the patient can add any of the fruits allowed. In addition, your patient occasionally drinks packaged fruit juices, which he should avoid. Also, some of the foods recorded by this participant are high in calorie content, such as avocados and "fried" foods such as squid, eggs or fried potatoes. The participant does not eat bread. We recommend asking him whether he believes bread to be fattening. If so, it should be explained to him that (for example) cooking and consuming fried foods is much more fattening since some of the frying oil is also present in the food he eats.
Note that at mid-morning and mid-afternoon your patient has coffee or yogurt, obviously with added sugar. Advise the participant to eat something with a high protein content as noted in our recommended diet, instead of liquids or dairy-containing products. They will make the participant feel more full and able to go until the next meal without feeling so hungry
In addition, for men participating in the study we should consider his spouse or partner, who should attend appointments with him given that she is likely to be the one who prepares food at home, and is also more likely to be the one who does the shopping, to a greater or lesser extent. The foods noted above (juice, yogurt, canned pineapple) should not be purchased. If the patient refrains from adding sugar to his coffee, consumes no-fat 0% yogurt and avoids fried foods it will be easier for him to comply with the 2000 kcal/day diet we recommend.
The physical activity done by this patient, due to his employment (gardener) is more than adequate. He should be advised that when he is on vacation he should take a ½-hour walk daily, at least 5 days per week.
