# Supplementary material for: Effectiveness of a low-fructose and/or low-sucrose diet in decreasing insulin resistance (DISFRUTE study): study protocol for a randomized controlled trial
Source: Trials. 2017 Aug 7;18:369. doi: 10.1186/s13063-017-2043-z (PMC5547514; doi:10.1186/s13063-017-2043-z)
Supplement: Supplementary file 4 — Case Record Form. (DOCX 74 kb) [file 13063_2017_2043_MOESM4_ESM.docx]

**NOMBRE:**

**CIP:**

**PACIENTE Nº:**

**TELEFONOS DE CONTACTO:**

**CENTRO DE SALUD:**

**CIAS:**

**PROFESIONAL:**

**FECHA PRIMER REGISTRO:**

**FECHA SEM 0, DÍA DE ANALITICA:**

**CAPTACION Y SEGUIMIENTO DEL PACIENTE**

**CIAS:**

**NOMBRE PACIENTE:**

**CAPTACION**

**BREVE CONSULTA MOTIVACIONAL** (hacer estas preguntas antes de informarle del estudio y pedirle que firme el consentimiento informado)

1.- ¿es para usted importante perder peso?

□ SI

□ NO

2.- ¿Conoce y asume usted los riesgos que tiene la obesidad para su salud?

□ SI

□ NO

3**.-¿**Es usted capaz de cambiar/seguir la dieta que le aconsejen los médicos o enfermeros si es incluido en un estudio para bajar de peso?

□ SI

□ NO

La respuesta negativa a una de estas tres preguntas lo incapacita para ser incluido en el estudio. En ese caso NO IMPRIMIR EL CONSENTIMIENTO INFORMADO. Si las tres respuestas son positivas y no tiene criterios de exclusión, el paciente puede ser incluido en el estudio, pero deberemos brevemente explicarle en qué consiste para que firme el consentimiento.

**1) CLASE SOCIAL:**

**1.-A) Estudios**

□ No sabe leer

□ Primarios completos o incompletos

□ Secundarios completos o incompletos

□ Universitarios completos o incompletos

**1.-B) *Renta familiar per capita (euros mensuales)**

□ ≤ 240

□ De 241 a 370

□ De 371 a 470

□ De 471 a 649

□ ≥ 650

*****(cociente entre el promedio de ingresos mensuales obtenidos por todos los miembros de la familia y el número de miembros de la misma).

**1.-C) **Índice de hacinamiento**

□ Más de 2 personas por dormitorio

□ Entre 1 y 2 personas por dormitorio

□ 1 persona por dormitorio

**(cociente entre el número de personas que habita el hogar y el número de dormitorios disponibles)

**1.-D) Estado civil**

□ Soltero

□ Casado o vive en pareja

□ Viudo /a,

□ Separado/a, divorciado/a

**2) HIPERTENSO**

□ SI

□ NO

**3) HIPERCOLESTEROLEMICO Y/O HIPERTRIGLICERIDEMICO**

□ SI

□ NO

**4) FUMADOR**

□ SI

□ NO

□ EX FUMADOR (DE MENOS DE UN AÑO)

□ EXFUMADOR (DE MAS DE UN AÑO)

5) Si fumador: ¿CUANTOS CIGARRILLOS , PUROS O PIPAS FUMA AL DIA?

Nº :

6) Su padre, su madre o alguno de sus hermanos ¿han padecido o padecen de diabetes?

□ SI

□ NO

7) Si la respuesta anterior fue SI, ¿podría indicarnos a qué edad se le diagnosticó?

Padre ____ años.

Madre ____ años.

Hermano/a 1 ____ años

Hermano/a 2 ____ años

Hermano/a 3 ____ años

**SEGUIMIENTO**

**FECHA (el día anterior o muy próximo a la extracción sanguínea):**

**Médico o enfermero que atiende al paciente:**

Talla: ­­­______ cms Peso: ______ kgs Cintura abdominal: ______ cms

Tensión Arterial 1: ______/_____ Tensión arterial 2:_____/_____

CUESTIONARIO DE ACTIVIDAD FISICA

¿Podría decirnos qué actividad física hace habitualmente en su trabajo?

**CUESTIONARIO DE ACTIVIDAD FÍSICA EN EL TIEMPO LIBRE**

A continuación encontrará dos páginas cada una con el mismo listado de actividades físicas y unas columnas para anotar el tipo y la actividad y posteriormente unos recuadros con la opción y con períodos de tiempo de realización de las mismas. La primera página se refiere a la actividad realizada la última semana y la segunda a la realizada los seis últimos meses.

La forma de rellenar el cuestionario es la siguiente:

1. Se lee atentamente cada actividad una a una y cuando se encuentre una que se haya realizado durante la última semana, con números claros y sin salirse del recuadro, se rellena en opción el número de la actividad correspondiente. Si existiese una quinta actividad rellenar a mano una quinta opción
2. Seguidamente se repite la misma acción para los últimos 6 meses. Ha de tener en cuenta que si ha realizado alguna actividad la última semana supone también que la ha realizado en los últimos 6 meses.

Para asegurar la uniformidad de la información recogida consideramos que:

- cada piso de escaleras = 1/2 min.
- una vuelta en esquí acuático = 5 min.
- un set de tenis individual = 20 min.
- un set de tenis dobles = 15 min.
- golf 9 hoyos = 90 min.

Ejemplo:

Una persona que:

- durante la última semana ha ido a caminar media hora cada día menos el fin de semana, tiene que anotar la opción 1, luego un 5 en la columna de días y 30 en minutos/día de práctica. Si durante los seis últimos meses también ha ido a caminar pero durante 2 meses no ha hecho esta actividad, tendrá que anotar 120 en la casilla de días de práctica al año 30 la casilla de minutos..
- durante la última semana ha subido 2 veces al día 2 pisos por la escalera, tiene que anotar en opción uno un 5, un 7 en la casilla de días de práctica, y un 2 en minutos. Si esta actividad la repite durante los últimos seis meses tendrá que anotar 180 en la casilla de días de la página siguiente y un 2 en la casilla de minutos.

**Sin ser la actividad que le exige su trabajo ¿Hizo algún tipo de actividad física o deporte en LA ULTIMA SEMANA (los siete días previos a hoy)? SI** □ **NO** □

**MARCAR LAS CUATRO MAS FRECUENTES**

**Andar - Bailar - Subir escaleras**

1.-Pasear

2 -Andar de casa al trabajo y del trabajo a casa

3.-Andar (llevando carrito de la compra)

4.-Andar (llevando bolsas de la compra)

5.-Subir escaleras

6.-Andar campo a través montaña

7.-Excursiones con mochila

8 -Escalar montañas

9.-Ir en bicicleta al trabajo

10.-Bailar

11.-Aerobic o ballet

12.-Jugar con los niños (corriendo, saltando,...)

**Ejercicios de mantenimiento general**

13.-Hacer ejercicio en casa

14.-Hacer ejercicio en un gimnasio

15.-Caminar deprisa

16.-Trotar ("Jogging")

17.-Correr 8 - 11 km/h

18.-Correr 12 - 16 km/h

19.-Levantar pesas

**Actividades acuáticas**

20.-Esquí acuático

21.-Surf

22.-Navegar a vela

23.-Ir en canoa o remar (por distracción)

24.-Ir en canoa o remar (en competición)

25.-Hacer un viaje en canoa

26.-Nadar (más de 150 metros en piscina)

27.-Nadar en el mar

28.- Bucear

**Deportes de invierno**

29.-Esquiar

30.-Esquí de fondo

31.-Patinar (ruedas o hielo)

**Otras actividades**

32.-Montar a caballo

33.-Jugar a los bolos

34.-Balonvolea

35.-Tenis de mesa

36.-Tenis individual

37.-Tenis dobles

38.-Badminton

39.-Baloncesto (sin jugar partido)

40.-Baloncesto jugando un partido)

41.-Baloncesto (actuando de árbitro)

42.-Squash

43.-Fútbol

44.-Golf (llevando el carrito)

45.-Golf (andando y llevando los palos)

46.-Balonmano

47.-Petanca

48.-Artes Marciales

49.-Motociclismo

50.-Ciclismo de carretera o montaña

**Actividades en el jardín**

51.-Cortar el césped con máquina

52.-Cortar el césped manualmente

53.-Limpiar y arreglar el jardín

54.-Cavar el huerto

55 -Quitar nieve con pala

**Trabajos y actividades caseras**

56.-Trabajos de carpintería dentro de casa

57.-Trabajos de carpintería (exterior)

58.-Pintar dentro de casa

59.-Pintar fuera de casa

60.-Limpiar la casa

61.-Mover muebles

**Caza y pesca**

62. -Tiro con pistola

63.-Tiro con arco

64.-Pescar en la orilla del mar

65.-Pescar con botas altas dentro del río

66 -Caza menor

67 -Caza mayor (ciervos, osos...)

**Otra actividad no mencionada** (escriba cuál o cuáles)

68.-………………………………………………

69.-………………………………………………

70.-………………………………………………

**OPCIÓN 1 OPCIÓN 2 OPCIÓN 3 OPCIÓN 4**

**¿Cuántas días practicó esa actividad durante la semana y cuántos minutos cada día?**

**OPCIÓN 1 días min/día**

**OPCIÓN 2 días min/día**

**OPCIÓN 3 días min/día**

**OPCIÓN 4** **días min/día**

**Sin ser la actividad que le exige su trabajo ¿Qué actividad física o deporte realizó en LOS ULTIMOS SEIS MESES?**

**MARCAR LAS CUATRO MAS FRECUENTES**

**Andar - Bailar - Subir escaleras**

1.-Pasear

2 -Andar de casa al trabajo y del trabajo a casa

3.-Andar (llevando carrito de la compra)

4.-Andar (llevando bolsas de la compra)

5.-Subir escaleras

6.-Andar campo a través montaña

7.-Excursiones con mochila

8 -Escalar montañas

9.-Ir en bicicleta al trabajo

10.-Bailar

11.-Aerobic o ballet

12.-Jugar con los niños (corriendo, saltando,...)

**Ejercicios de mantenimiento general**

13.-Hacer ejercicio en casa

14.-Hacer ejercicio en un gimnasio

15.-Caminar deprisa

16.-Trotar ("Jogging")

17.-Correr 8 - 11 km/h

18.-Correr 12 - 16 km/h

19.-Levantar pesas

**Actividades acuáticas**

20.-Esquí acuático

21.-Surf

22.-Navegar a vela

23.-Ir en canoa o remar (por distracción)

24.-Ir en canoa o remar (en competición)

25.-Hacer un viaje en canoa

26.-Nadar (más de 150 metros en piscina)

27.-Nadar en el mar

28.- Bucear

**Deportes de invierno**

29.-Esquiar

30.-Esquí de fondo

31.-Patinar (ruedas o hielo)

**Otras actividades**

32.-Montar a caballo

33.-Jugar a los bolos

34.-Balonvolea

35.-Tenis de mesa

36.-Tenis individual

37.-Tenis dobles

38.-Badminton

39.-Baloncesto (sin jugar partido)

40.-Baloncesto jugando un partido)

41.-Baloncesto (actuando de árbitro)

42.-Squash

43.-Fútbol

44.-Golf (llevando el carrito)

45.-Golf (andando y llevando los palos)

46.-Balonmano

47.-Petanca

48.-Artes Marciales

49.-Motociclismo

50.-Ciclismo de carretera o montaña

**Actividades en el jardín**

51.-Cortar el césped con máquina

52.-Cortar el césped manualmente

53.-Limpiar y arreglar el jardín

54.-Cavar el huerto

55 -Quitar nieve con pala

**Trabajos y actividades caseras**

56.-Trabajos de carpintería dentro de casa

57.-Trabajos de carpintería (exterior)

58.-Pintar dentro de casa

59.-Pintar fuera de casa

60.-Limpiar la casa

61.-Mover muebles

**Caza y pesca**

62. -Tiro con pistola

63.-Tiro con arco

64.-Pescar en la orilla del mar

65.-Pescar con botas altas dentro del río

66 -Caza menor

67 -Caza mayor (ciervos, osos...)

**Otra actividad no mencionada** (escriba cuál o cuáles)

68.-………………………………………………

69.-………………………………………………

70.-………………………………………………

**OPCIÓN 1 OPCIÓN 2 OPCIÓN 3 OPCIÓN 4**

**¿Cuántas días practicó esa actividad EN LOS ULTIMOS SEIS MESES y cuántos minutos cada/día?**

**OPCIÓN 1 días min/día**

**OPCIÓN 2 días min/día**

**OPCIÓN 3 días min/día**

**OPCIÓN 4** **días min/día**

**Paciente: CIP**

**FECHA (semana 2):**

**Profesional que atiende al paciente:**

Inicio del consejo dietético específico y consejos generales sobre actividad física (si hace una actividad superior a caminar 150 min /sem (unos 30 min 5 días a la semana), animarle a seguir haciéndola. Si hace menos actividad o ninguna, comprobar que no hay contraindicaciones para realizar actividad física y recomendarle realizar 30 min/día de actividad 5 días a la semana.

**FECHA (semana 4):**

**Profesional que atiende al paciente:**

Peso: ______ kgs Cintura abdominal: ______ cms

Tensión Arterial 1: ______/_____ Tensión arterial 2:_____/_____

Reforzamiento consejo dietético.

**FECHA (semana 8):**

**Profesional que atiende al paciente:**

Peso: ______ kgs Cintura abdominal: ______ cms

Tensión Arterial 1: ______/_____ Tensión arterial 2:_____/_____

HACER RECORDATORIO DE 24 HORAS (para valorar la adherencia al tratamiento)

Una vez finalizado el recordatorio realizar intervención dietética.

**FECHA (semana 12):**

**Profesional que atiende al paciente:**

Peso: ______ kgs Cintura abdominal: ______ cms

Tensión Arterial 1: ______/_____ Tensión arterial 2:_____/_____

HACER RECORDATORIO DE 24 HORAS

Reforzar intervención dietética.

**Paciente: CIP**

**FECHA (semana 16):**

**Profesional que atiende al paciente:**

Peso: ______ kgs Cintura abdominal: ______ cms

Tensión Arterial 1: ______/_____ Tensión arterial 2:_____/_____

HACER RECORDATORIO DE 24 HORAS

Reforzar intervención dietética.

**FECHA (semana 20):**

**Profesional que atiende al paciente:**

Peso: ______ kgs Cintura abdominal: ______ cms

Tensión Arterial 1: ______/_____ Tensión arterial 2:_____/_____

HACER RECORDATORIO DE 24 HORAS

Reforzar intervención dietética.

SEMANA 21-22: Entregar al paciente el mismo dossier que en la semana -2 para que lo lleve en la semana 24.

**FECHA (semana 24)** (el día anterior o muy próximo a la extracción sanguínea)

**Profesional que atiende al paciente:**

REVISAR con el paciente los registros de los 4 días y hacer la encuesta de actividad física.

Peso: ______ kgs Cintura abdominal: ______ cms

Tensión Arterial 1: ______/_____ Tensión arterial 2:_____/_____

**EFECTOS ADVERSOS DE LA DIETA**: estimado participante ¿podría decirnos si durante el periodo del estudio, y como consecuencia de la dieta, ha sentido algún efecto adverso?

□ SI Si la respuesta ha sido SI, ¿podría decirnos cuál/es?:

□ NO

B) Seguir esta dieta ¿qué grado de esfuerzo le ha supuesto?

⁬ 1.-Ningún esfuerzo

⁬ 2.-Poco esfuerzo

⁬ 3.-Bastante esfuerzo

⁬ 4.-Muchísimo esfuerzo

……………………………………………………………………………………..

**Paciente: CIP**

**FECHA (semana 48)** (el día anterior o muy próximo a la extracción sanguínea)

**Profesional que atiende al paciente:**

Peso: ______ kgs Cintura abdominal: ______ cms

Tensión Arterial 1: ______/_____ Tensión arterial 2:_____/_____

SEMANA 8. FECHA:

**NOMBRE: NºHIST:**

**fee**

Preguntar al paciente lo que comió y bebió el día anterior con el máximo detalle en cuanto a tipo de alimento, bebida y cantidades y raciones.

**Desayuno**

**Media**

**mañana**

**Almuerzo**

**Cena**

**Antes de**

**Acostarse**

**Otras**

**Comidas**

**entre horas**

**Merienda**

SEMANA 12 . FECHA:

**NOMBRE: NºHIST:**

**fee**

Preguntar al paciente lo que comió y bebió el día anterior con el máximo detalle en cuanto a tipo de alimento, bebida y cantidades y raciones.

**Desayuno**

**Media**

**mañana**

**Almuerzo**

**Cena**

**Antes de**

**Acostarse**

**Otras**

**Comidas**

**entre horas**

**Merienda**

SEMANA 16. FECHA:

**NOMBRE: Nº HIST:**

**fee**

Preguntar al paciente lo que comió y bebió el día anterior con el máximo detalle en cuanto a tipo de alimento, bebida y cantidades y raciones.

**Desayuno**

**Media**

**mañana**

**Almuerzo**

**Cena**

**Antes de**

**Acostarse**

**Otras**

**Comidas**

**entre horas**

**Merienda**

SEMANA 20. FECHA:

**NOMBRE: NºHIST:**

**fee**

Preguntar al paciente lo que comió y bebió el día anterior con el máximo detalle en cuanto a tipo de alimento, bebida y cantidades y raciones.

**Desayuno**

**Media**

**mañana**

**Almuerzo**

**Cena**

**Antes de**

**Acostarse**

**Otras**

**Comidas**

**entre horas**

**MeriendaA LAS 24 SEMANAS**

**Sin ser la actividad que le exige su trabajo ¿Hizo algún tipo de actividad física o deporte en LA ULTIMA SEMANA (los siete días previos a hoy)? SI □ NO** □

**MARCAR LAS CUATRO MAS FRECUENTES**

**Andar - Bailar - Subir escaleras**

1.-Pasear

2 -Andar de casa al trabajo y del trabajo a casa

3.-Andar (llevando carrito de la compra)

4.-Andar (llevando bolsas de la compra)

5.-Subir escaleras

6.-Andar campo a través montaña

7.-Excursiones con mochila

8 -Escalar montañas

9.-Ir en bicicleta al trabajo

10.-Bailar

11.-Aerobic o ballet

12.-Jugar con los niños (corriendo, saltando,...)

**Ejercicios de mantenimiento general**

13.-Hacer ejercicio en casa

14.-Hacer ejercicio en un gimnasio

15.-Caminar deprisa

16.-Trotar ("Jogging")

17.-Correr 8 - 11 km/h

18.-Correr 12 - 16 km/h

19.-Levantar pesas

**Actividades acuáticas**

20.-Esquí acuático

21.-Surf

22.-Navegar a vela

23.-Ir en canoa o remar (por distracción)

24.-Ir en canoa o remar (en competición)

25.-Hacer un viaje en canoa

26.-Nadar (más de 150 metros en piscina)

27.-Nadar en el mar

28.- Bucear

**Deportes de invierno**

29.-Esquiar

30.-Esquí de fondo

31.-Patinar (ruedas o hielo)

**Otras actividades**

32.-Montar a caballo

33.-Jugar a los bolos

34.-Balonvolea

35.-Tenis de mesa

36.-Tenis individual

37.-Tenis dobles

38.-Badminton

39.-Baloncesto (sin jugar partido)

40.-Baloncesto jugando un partido)

41.-Baloncesto (actuando de árbitro)

42.-Squash

43.-Fútbol

44.-Golf (llevando el carrito)

45.-Golf (andando y llevando los palos)

46.-Balonmano

47.-Petanca

48.-Artes Marciales

49.-Motociclismo

50.-Ciclismo de carretera o montaña

**Actividades en el jardín**

51.-Cortar el césped con máquina

52.-Cortar el césped manualmente

53.-Limpiar y arreglar el jardín

54.-Cavar el huerto

55 -Quitar nieve con pala

**Trabajos y actividades caseras**

56.-Trabajos de carpintería dentro de casa

57.-Trabajos de carpintería (exterior)

58.-Pintar dentro de casa

59.-Pintar fuera de casa

60.-Limpiar la casa

61.-Mover muebles

**Caza y pesca**

62. -Tiro con pistola

63.-Tiro con arco

64.-Pescar en la orilla del mar

65.-Pescar con botas altas dentro del río

66 -Caza menor

67 -Caza mayor (ciervos, osos...)

**Otra actividad no mencionada** (escriba cuál o cuáles)

68.-………………………………………………

69.-………………………………………………

70.-………………………………………………

**OPCIÓN 1 OPCIÓN 2 OPCIÓN 3 OPCIÓN 4**

**¿Cuántas días practicó esa actividad durante la semana y cuántos minutos cada día?**

**OPCIÓN 1 días min/día**

**OPCIÓN 2 días min/día**

**OPCIÓN 3 días min/día**

**OPCIÓN 4** **días min/día**

**Sin ser la actividad que le exige su trabajo ¿Qué actividad física o deporte realizó en LOS ULTIMOS SEIS MESES?**

**MARCAR LAS CUATRO MAS FRECUENTES**

**Andar - Bailar - Subir escaleras**

1.-Pasear

2 -Andar de casa al trabajo y del trabajo a casa

3.-Andar (llevando carrito de la compra)

4.-Andar (llevando bolsas de la compra)

5.-Subir escaleras

6.-Andar campo a través montaña

7.-Excursiones con mochila

8 -Escalar montañas

9.-Ir en bicicleta al trabajo

10.-Bailar

11.-Aerobic o ballet

12.-Jugar con los niños (corriendo, saltando,...)

**Ejercicios de mantenimiento general**

13.-Hacer ejercicio en casa

14.-Hacer ejercicio en un gimnasio

15.-Caminar deprisa

16.-Trotar ("Jogging")

17.-Correr 8 - 11 km/h

18.-Correr 12 - 16 km/h

19.-Levantar pesas

**Actividades acuáticas**

20.-Esquí acuático

21.-Surf

22.-Navegar a vela

23.-Ir en canoa o remar (por distracción)

24.-Ir en canoa o remar (en competición)

25.-Hacer un viaje en canoa

26.-Nadar (más de 150 metros en piscina)

27.-Nadar en el mar

28.- Bucear

**Deportes de invierno**

29.-Esquiar

30.-Esquí de fondo

31.-Patinar (ruedas o hielo)

**Otras actividades**

32.-Montar a caballo

33.-Jugar a los bolos

34.-Balonvolea

35.-Tenis de mesa

36.-Tenis individual

37.-Tenis dobles

38.-Badminton

39.-Baloncesto (sin jugar partido)

40.-Baloncesto jugando un partido)

41.-Baloncesto (actuando de árbitro)

42.-Squash

43.-Fútbol

44.-Golf (llevando el carrito)

45.-Golf (andando y llevando los palos)

46.-Balonmano

47.-Petanca

48.-Artes Marciales

49.-Motociclismo

50.-Ciclismo de carretera o montaña

**Actividades en el jardín**

51.-Cortar el césped con máquina

52.-Cortar el césped manualmente

53.-Limpiar y arreglar el jardín

54.-Cavar el huerto

55 -Quitar nieve con pala

**Trabajos y actividades caseras**

56.-Trabajos de carpintería dentro de casa

57.-Trabajos de carpintería (exterior)

58.-Pintar dentro de casa

59.-Pintar fuera de casa

60.-Limpiar la casa

61.-Mover muebles

**Caza y pesca**

62. -Tiro con pistola

63.-Tiro con arco

64.-Pescar en la orilla del mar

65.-Pescar con botas altas dentro del río

66 -Caza menor

67 -Caza mayor (ciervos, osos...)

**Otra actividad no mencionada** (escriba cuál o cuáles)

68.-………………………………………………

69.-………………………………………………

70.-………………………………………………

**OPCIÓN 1 OPCIÓN 2 OPCIÓN 3 OPCIÓN 4**

**¿Cuántas días practicó esa actividad EN LOS ULTIMOS SEIS MESES y cuántos minutos cada/día?**

**OPCIÓN 1 días min/día**

**OPCIÓN 2 días min/día**

**OPCIÓN 3 días min/día**

**OPCIÓN 4** **días min/día**

**Paciente: CIP**

**PACIENTE QUE ABANDONA EL SEGUIMIENTO:**

□ SI

□ NO

**CAUSAS DEL ABANDONO**

□ NO LE INTERESA EL ESTUDIO

□ TRASLADO DE RESIDENCIA

□ TEMPORALMENTE FUERA DEL AREA

□ DIFICULTAD PARA SEGUIR LA DIETA

□ ILOCALIZABLE

□ FALTA DE TIEMPO

□ FALLECIMIENTO (causa: )

□ LE ACONTECE DIABETES, UNA ENFERMEDAD GRAVE O INCAPACITANTE (ESPECIFICAR: )

□ OTRAS CAUSAS: (ESPECIFICAR: )
