# Supplementary material for: Effectiveness of a low-fructose and/or low-sucrose diet in decreasing insulin resistance (DISFRUTE study): study protocol for a randomized controlled trial
Source: Trials. 2017 Aug 7;18:369. doi: 10.1186/s13063-017-2043-z (PMC5547514; doi:10.1186/s13063-017-2043-z)
Supplement: Supplementary file 6 — Foods included to date and sources of macronutrients and sugars (Spanish). (DOCX 35 kb) [file 13063_2017_2043_MOESM6_ESM.docx]

| ALIMENTO |  | **Referencia Mataix** | **Nombre en base de datos-alimentos o promedio de alimentosOBSERVACIONES** |
| --- | --- | --- | --- |
| PAN DE MOLDE NO INTEGRAL (rebanada = 20 gr ) | c1 | 71 | pan de molde BIMBO DOS |
| PAN DE MOLDE INTEGRAL (rebanada= 20 gr ) | c2 | 74 |  |
| PAN NORMAL NO INTEGRAL (gramos) | c3 | 68 | pan blanco |
| PAN NORMAL INTEGRAL (gramos) | c4 | 78 | pan integral de trigo |
| GOFIO (cuchara sopera= 20 gr) | c5 | 46 ; 47 | Promedio Trigo -Maiz |
| CEREALES AZUCARADOS (cucharones= 30 g) | c6 | 30 ; 27;31;32,63, 87,89 | Promedio: copos de maiz azucarados (kellogs) copos maíz azucarados pascual, arroz con miel pascual,maiz tostaods miel y cacahuetes kellogs, maiz inflado con miel, trigo inflsdo con miel, trigo inflado y azucarado |
| CEREALES SIN AZUCAR (cucharones= 30g) | c7 | 13,14, 28 ;29; 33 | Promedio: arroz tostado inflado pascual, arroz tostadoinflado kellosgs, copos de maíz tostados corn flakes (kellogs) , maiz tostado (pascual) , copos arroz y trigo special K |
| PAPAS FRITAS (gramos) (pag 92) | c8 |  | para 100 gr 30 ml de aceite de oliva. Elaboración propia. |
| PAPAS GUISADAS (unidades=60 g_pag 28) | c9 | 221 | patata hervida, aplicada la porción comestible |
| PAN TOSTADO (CLÁSICO E NTEGRAL ) (Rebanadas = 11 g) | c10 |  | composición tomada de envoltorio (marca hacendado) |
| CARNE COCHINO, COSTILLAS, NO CONTAR EMBUTIDOS, | c11 | 582;584 586; 594 | chuleta, costillas, lomo, solomillo |
| CARNE VACA, TERNERA, BUEY, BECERRO gr | c12 | 605 ; 614; 618 | carne magra de ternera , solomillo de ternera ; solomillo de Buey |
| CARNE CONEJO, LIEBRE gr | c13 | 624 | conejo |
| CARNE POLLO, AVES gr | c14 | 629 ; 630; 631 ; 636 ; 637 | gallina, pato, muslo de pavo, muslo de pollo, pechuga de pollo |
| JAMON SERRANO (lonchas = 50 g) , (si son finas, media loncha) | c15 | 650 |  |
| JAMON COCIDO DEL EXTRA y NORMAL (lonchas= 20 g) | c16 | 649 | jamón cocido |
| PECHUGA DE PAVO (lonchas= 20 g) | c17 | 661 | pechuga de pavo |
| HIGADO FRESCO (ASADURA) (gramos) (similar albondigas | c18 | 585; 599; 609; 616; 635 | hígado de cerdo, cordero, ternera, buey, pollo |
| CARNE CORDERO (gramos) | c19 | 596 ; 601 | chuleta y pierna de cordero |
| HUEVOS (UNIDAD=50g) | c20 | 756 | huevo de gallina |
| HAMBURGUESAS (CON SU PANECILLO), UNIDAD = 185 g | c21 | 80, 1028, 1021, 1015 | 60g panecillo, 10g Mostaza, 15g Ketchup |
| PERRITOS CALIENTES (CON SU PANECILLO), unidad = 120 g | c22 | 664,80,1021,1015 | 45g panecillo, 50g salchicha, 10g mostaza, 15 g Ketchup |
| PESCADO BLANCO(SAMA, VIEJA, ALFONSINA, BESUGO, CHERNE) (gramos) | c23 | 668 ; 687;689;693,696,697,711,714 | abadejo, dorada, gallo, lenguado, merluza, mero, sargo, vieja |
| PESCADO NEGRO O AZUL (ATÚN, SARDINA, CABALLA, BURRO, SARGO, CHOPAS) (gramos) 5 | c24 | 673;678, 692, 710; 681 | atún, bonito, caballa, jurel, sardina |
| PULPO, CHOCO, POTA, CALAMARES (gramos) | c25 | 716,727 | calamares, pulpo |
| MARISCO (LAPAS, GAMBAS, CENTOLLOS) (gramos) | c26 | 715,717,719,721,722,724 | almeja, chirla, berberecho, cangrejos, nécoras, centollo, gamba y camarón, langosta y bovagante, mejillón |
| PESCADO SALADO O SECO (TOLLOS, JAREAS, POTAS…) gramos | c27 | 732, 733, 737, 738, 748 | arenque ahumado, arenque salado, bacalao ahumado, bacalao salado, salmón ahumado |
| SALCHICHAS (gramos ) | c28 | 663, 664, 665 | salchichas frescas, frankfurt, tipo Viena |
| VISCERAS (CALLOS, RIÑONES) (gramos) | c29 | 583,591,597,602,603,604,607,612,613 | (callos, riñones, corazón, sesos)cerdo, cordero y ternera |
| TOCINO, BACON, CHICHARRONES (Loncha= 18gr) | c30 | 595, 638, 642 | Tocino, bacon, chicharrones |
| CHORIZO CANARIO DE UNTAR (UNIDADES= 40gr) | c31 | 667 | sobrasada |
| CHORIZO U OTROS EMBUTIDOS DE CORTAR EN RODAJAS (LONCHAS = 20 gr) | c32 | 643, 644, 645, 648, 653, 656, 662 | chopped de cerdo, chopped de pavo, chorizo, fuet primera, lomo embuchado, mortadela, salami |
| PARAGUAYO ( Gramos/ pieza= 65g pequeño,100g med o 135g grande ) | c33 |  | Base BEDCA: para azúcares, equivalencias a proporciones de melocotón. (sacarosa 7 gr, fructosa 1,6, glucosa 1,6) |
| ENSALADA (Gramos) | c34 | 194, 217,222,226,232,236,238, 239, 242,768, 839 | Plato compuesto: cebolla, lechuga, pepino, pimiento, tomate crudo, zanahoria, aceituna negra y verde,aguacate, aceite de oliva, vinagre |
| MANZANA (GRAMOS) | c35 | 269, 270, 271 | manzana roja, golden, granny smith |
| PERA (GRAMOS ) | c36 | 280 | pera |
| PLATANO (GRAMOS ) | c37 | 282 | plátano |
| NARANJA, MANDARINA,POMELO, LIMON (GRAMOS ) | c38 | 265,267,276,283 | limón , mandarina, pomelo, naranja |
| PAPAYAS (GRAMOS_ COMPARATIVO TROZO MELON ) | c39 | 279 | Papaya |
| UVAS ( LO QUE CABE EN UNA MANO O UN BOL) (EQUIV A 200 GRAMOS) ( uva pequeña=6g), (uva grande=12g) | c40 | 289, 290 | uva blanca y negra |
| JUGO NATURAL (recién hecho, no envasado) (ml) | c41 | 299 , 307 | Jugo natural recién hecho limón y naranja |
| ZUMO DE FRUTAS ( ENVASADO) (ML) | c42 | 296,302,304,305 | zumo multifrutas (kasfruit), manzana ( kasfruit), melocotón y uva (kasfruit), naranja(comercial) |
| FRUTAS ENVASADAS (ALMIBAR) (GRAMOS) | c43 | 314, 320, 329 | cóctel de frutas en almibar, melocotón en almibar, piña en almíbar |
| TOMATE CRUDO ( PIEZAS=145gr ) | c44 | 232 | tomate |
| AGUACATE (PIEZAS) ESTIMADO 150 GRAMOS /PIEZA) |  |  |  |
| ACEITUNAS (UNIDADES) | c46 | 238, 239 | aceituna negra y verde |
| CEBOLLAS CRUDAS (PIEZAS) (ESTIMADO 110 GRAMOS /PIEZA) | c47 | 194 | cebolla cruda |
| PIMIENTOS CRUDOS (ROJOS O VERDES, ESTIM PIEZA 120 GR) | c48 | 226 | pimiento crudo |
| PEPINO (PIEZA (ESTIMADO 150 GRAMOS) | c49 | 222 | pepino |
| MANGOS/AS (PIEZA, ESTIMADO 230 GRAMOS) | c50 | 268 | mangosy mangas |
| KIWI (PIEZA_ ESTIMADO 90 GRAMOS) | c51 | 262 | kiwi |
| HIGOS FRESCOS (PIEZAS, ESTIMADO 50 GRAMOS) | c52 bis | 259 | higo y breva |
| TUNOS, HIGOS CHUMBOS (PIEZAS; ESTIMADO 60 GRAMOS) | c52 | 260 | higo chumbo |
| LECHUGA ( GRAMOS ) | c53 | 217 | lechuga |
| AJOS CRUDOS O NO (DIENTES) | c54 | 179 | ajo |
| LECHE EN POLVO (BEBIDA) ML | c55 | 455 | leche en polvo |
| LECHE DE CABRA ML | c56 | 428 | leche de cabra |
| LECHE ENTERA LIQUIDA ML | c57 | 445 | leche entera vaca |
| LECHE DESNATADA LIQUIDA ML | c58 | 439 | leche desnatada UHT |
| LECHE SEMIDESNATADA LIQUIDA ML (y el DANACOL = 100 ML) | c59 | 461 | leche semidesnatada UHT (incluir el danacol). |
| LECHE CON GRASA VEGETAL (MILLAC, PREPARADOS LACTEOS) ML | c60 | 435 438 | leche desnatada y grasa vegetal de oliva(Lillac) leche desnatada reconstituida flora con aceites vegetales |
| LECHE CONDENSADA (CUCHARAS DE POSTRE= 10 g) | c61 | 430 | leche condensada |
| LECHE DE SOJA (ML) | c62 |  | composición azúcares tomada de envoltorio (marca hacendado) Resto MATAIX. |
| LECHE DE SOJA CHOCOLATE (ML) | c63 |  | composición azúcares tomada de envoltorio (marca hacendado) |
| PETIT SUISSE (VASITOS=60gr) | c64 | 486, 489, 490, 493 | pettir suisse desnatado con frutas DANONE, con frutas y cereales DANONE, natural azucarado DANONE; sabores DANONE |
| FLANES, NATILLAS (INDUSTRIALES) (VASITOS 140) | c65 | 410, 413, 476, 479 | flan de huevo DANONE; flan de vainilla DANONE; natilla de chocolate DANONE; natilla de vainilla NESTLE |
| BATIDOS INDUSTRIALES DE LECHE (ML) | c66 | 367, 369, 373, 375 | batido de cacao PULEVA; chocolate bajo en calorías Mc Donalds, vainilla bajo en calorías McDonalds, vainilla UHT PULEVA |
| NATA,CREMA DE LECHE (ML) | c67 | 470, 473, 474 | nata, nata pasteurizada PULEVA, nata UHT PULEVA |
| QUESO BLANCO TIERNO (gr) | c68 | 495, 506 | queso blanco desnatado, queso fresco desnatado DANONE, queso de Burgos ARIAS |
| QUESO SEMICURADO (AMARILLO O BLANCO) (gr) | c69 | 519, 523 | queso manchego semicurado, queso semicurado graso PULEVA |
| QUESO CURADO (AMARILLO O BLANCO) (gr) | c70 | 505, 517, 520 | queso emmental, manchego curado, parmesano |
| MANTEQUILLA (CUCHARA POSTRE _EQUIV 20 GRAMOS) | c71 | 776 | mantequilla sin sal |
| MARGARINA (CUCHARA POSTRE _EQUIIV 20 GRAMOS) | c72 | 780 | margarina |
| ACEITE DE OLIVA (CUCHARA POSTRE _5 GRAMOS) (PAG 49_CUCH SOPERA 10 GR) | c73 | 768 | aceite de oliva |
| OTRO ACEITE (CUCHARAS DE POSTRE) | c73bis | 762, 765, 767, 771 | aceite de cacahuete, girasol, maíz, soja |
| MAYONESA (CUCHARA DE POSTRE_ EQUIV 10 GRAMOS) | c74 | 783 | mayonesa comercial |
| KETCHUP Y OTRAS SALSAS DE TOMATE COMPRADAS (CUCHARAS POSTRE) | c75 | 1015, 1026, 1027 | ketchup, tomate natural tamizado (SOLIS), tomate frito ORLANDO |
| FRUTOS SECOS (MANICES, ALMENDRAS, NUECES) (CUCHARON_60 gr_LO QUE CABE EN LA MANO) | c76 | 348, 351, 352, 353, 356 | almendra, avellana, cacahuete, castaña, nuez |
| MOJO PICON, TABASCO (CUCHARAS DE POSTRE) | c77 | 179, 196 225, 768, 839 | ajo, chile rojo, pimienta negra, aceite de oliva, vinagre |
| MOSTAZA (CUCHARAS DE POSTRE= 10g) | c78 | 1016 | mostaza |
| AZUCAR (gr) | c79 | 131 | azúcar |
| MIEL (CUCHARAS DE POSTRE=10g) | c80 | 167 | miel |
| HELADOS (BOLAS DE HELADO=80gr) | c81 | 418 | helados Mataix (proporción de H-C: finish institute: food number 663: 69,3 % sacarosa y 30,7 % lactosa) |
| POLOS (UNIDADES=70g)o SORBETES | c82 |  | FINISH INSTITUTE: ALIMENTO Nº: 31991 Sorbete (Carte D'or) |
| GALLETAS TOSTADAS/MARÍA INTEGRAL (Unidades= 6g) | c83 |  | composición tomada de envoltorio (marca hacendado) |
| PASTELERIA CASERA (1 RODAJA DE BIZCOCHÓN) | c84 | 55, 131, 444 , 776, 837 | harina de trigo,azúcar, leche entera, huevo , mantequilla, levadura. Elaboración propia. |
| GALLETAS SIN CREMA (MARIA) Número de galletas | c85 | 118 | galletas María |
| GALLETAS CON CREMA, incluye galletas con chocolate | c86 | 113, 117, 120 | galletas chocolate, galletas digestive chocolate, napolitanas rellenas con crema de cacao DULCESOL |
| CAFÉ FILTRADO (CAFETERA ELECTRICA TIPO ALEMAN) (TACITAS)  c87 | |  | café expreso infusión |
| CAFÉ EXPRESO (CASERO O DE BARES Y CAFETERIAS)(mL) | c88 | 796 |  |
| CAFÉ DESCAFEINADO (mL) | c89 | 796 |  |
| CACAO AZUCARADO EN POLVO, (colacao= 20 g) | c90 | 793,792 | cacao en polvo azucarado, composición tomada de envoltorio (marca colacao), cacao soluble NESQUICK |
| CHOCOLATE, CHOCOLATINAS , BOMBONES (BARRITAS=40g) | c91 | 135, 136, 141, 144, 161, 162 | bombones, bombones caja roja NESTLE, chocolate, chocholate blanco, chocolatina extafino leche almendra NESTLE, chocolatina extafino negro almendra NESTLE |
| DULCE DE MEMBRILLO O DULCE DE GUAYABA (LONCHAS= 15 g) | c92 | 321 | membrillo y pastas de fruta |
| MERMELADA (CUCHARAS= 12 g) | c93 | 322,324,327 | mermelada de albaricoque, ciruela naranja |
| CARAMELOS Y OTRAS GOLOSINAS (UNIDADES=5g) | c94 | 138,14 | caramelos y chicles con azúcar, incluye chupetes y gominolas |
| HIGOS PASADOS (UNIDADES= 17 g) | c95 | 333 | higo desecado |
| PASAS (CUCHARÓN) | c95bis | 335 | uva pasa |
| SANDIA (RODAJAS) | c96 | 285 | sandía |
| MELON(RODAJAS) | c97 | 273 | melón |
| FRESAS (UNIDADES) | c98 | 254 | fresa y fresón |
| CEREZAS (UNIDADES) | c99 | 246 | cerezas |
| BERROS (CUCHARÓN) | c100 | 186 | berros |
| PIMIENTOS PICANTES (ENTEROS, NO DE MOJO) (UNIDADES) | c101 | 196, 197 | chile rojo, chile verde |
| ARROZ (COMO SEA EN CUALQUIER COMIDA)(GRAMOS) | c102 | 8, 9, 10, 11 | arroz integral crudo, integral hervido, pulido sin cáscara crudo, pulido sin cáscara hervido |
| VERDURA GUISADA (HABICHUELA, ZANAHORIA, CALABACIN, COL, BUBANGOS, CALABAZA, COLIFRLOR)(GRAMOS) | c103 | 190,192, 204, 206, 216, 237 | calabacín hervido, habichuelas, calabaza, zanahorias, col y repollo, coliflor |
| TORTITAS DE MAÍZ ( UNIDADES) | c104 |  | composición tomada de envoltorio (marca hacendado). Asumimos azúcar = sacarosa. Asumimos grasa mono y poli al 50 % y restando a las totales las satur que si vienen en envoltorio |
| CEREALES INTEGRALES (cucharones=30g) | c105 | 22,66 | All Bran KELLOGS, salvado de trigo HACENDADO, muesli KELLOGS |
| CEREALES CHOCOLATE (cucharones=30g) | c106 |  | Cereal relleno chocolate HACENDADO |
| TE (ML) | c107 | 826 | te |
| REFRESCOS LIGHT (ML) | c108 | 788 | bebida gaseosa light |
| REFRESCOS NO LIGHT CON GAS (ML) | c109 | 822 | refresco de cola |
| VINO, CAVA , CHAMPAN (ML) | c110 | 804,833 | cava, vino de mesa |
| CERVEZA, SIDRA (ML) | c111 | 805,806,824 | cerveza, cerveza negra, sidra |
| LICORES DULCES Y VINOS DULCES (ML) | c112 | 820, 821, 830, 831 | licor de café, licores dulces, vino dulce ( málaga), vino moscatel ( glassful) |
| RON Y COÑAC (ML) | c113 | 814,823 | ron, coñac |
| AGUARDIENTES, WHISKY (ML) | c114 | 786, 835 | aguardiente, whisky |
| AGUA DEL GRIFO (ML) | c115 |  |  |
| AGUA EMBOTELLADA CON GAS (ML) | c116 | 785,825 | agua mineral perrier, soda sifón |
| AGUA EMBOTELLADA SIN GAS (ML) | c117 | 784 | agua mineral de mesa |
| REFRESCOS SIN GAS (ML) | c117bis | 789, 790 | bebida isotónica aquarius, gatorade |
| SAL (CUCHARAS) | c118 |  | Dieta hiposódicaen www.fisterra.com. Dra María Carreira Miño |
| SARDINAS O ATUN EN LATA (LATAS=85g) | c119 | 734, 735, 749 | atún en aceite, atún enlatado en agua, sardinas en aceite |
| ÑAME, BONIATO, BATATAS (GRAMOS) | c120 | 183 | batata y boniato |
| INFUSIONES 1 (ML) | c121 |  | TCA del libro blanco de evaluación del estado nutricional de la población catalana 1992-93 LAROUSSE |
| SOPAS DE SOBRE (cucharon=100g) | c122 | 989,992, 994 , 998 , 999 | ave con arroz, pollo con fideos, ternera jardinera y maravilla. |
| PASTAS GUISADAS EN CASA (MACARRONES, SPAGUETIS, ETC) (GRAMOS) PÁG.20-21 | c123 | 38, 41, 42, 59, 61 | espagueti integral hervido, espagueti normal hervido con sal, espagueti normal hervido sin sal, lasaña hervida, macarrones hervidos |
| POTAJES, (CUCHARÓN) | c124 | 62, 176, 179, 191, 194, 189, 215, 220, 223, 232, 236, 608, 768 y 992 | maíz, acelga, ajo, calabaza, cebolla, calabacín, judía verde, patata, perejil, tomate crudo, zanahoria, costillas de ternera, aceite de oliva |
| COMIDA PREPARADA DE LATA (FABADA, ETC) (CUCHARÓN) | c125 | 921, 923, 934, 937 | callos a la madrileña (TILA), cocido español (LITORAL), fabada asturiana (LITORAL), lentejas con chorizo (LITORAL) |
| PIZZAS PRECOCINADas ( gramos) 1 entera=350 g, 1/8=44g | c126 | 895, 899 , 903 | PIZZA DE ATÚN, PIZZA DE JAMÓN Y QUESO, PIZZA MARGARITA QUESO Y TOMATE |
| RANCHO CANARIO, cucharón= 100g | c127 |  | Elaboración propia a partir de receta |
| SALPICÓN DE ATÚN | c128 |  | Elaboración propia a partir de receta |
| PATATAS CHIPS (PAG 92) | c129 | 1014 |  |
| CROISSANT , unidad= 100g | c130 | 104 |  |
| CROISSANT DE CHOCOLATE gr, unidad= 100g | c131 | 105 |  |
| MAGDALENAS, gramos | c132 | 119 |  |
| DONUTS NORMALES/CHOCOLATE unidad=65g | c133 | 107,108 | promedio donuts normales y de chocolate |
| polen de abeja cucharadas (cucharada postre = 10 gr) | c134 |  | composición envoltorio polen de abeja hacendado (azúcares: revista apicultura 1993; nº 59. Mayo) |
| JUDÍAS A LA VINAGRETA, gr | c135 |  | elaboración propia a partir de receta |
| Escaldón de Gofio (una ración = 150 gramos) | C136 |  | receta canaria a partir de sus ingredientes. (una ración = 150 gramos) no incluye el MOJO |
| tortilla de papas (gramos) | c137 |  | elaboración propia a partir de receta |
| Garbanzos Compuestas (cucharón = 100gr) | c138 |  | elaboración propia a partir de receta; aquí incluímos la ropa vieja. |
| huevo frito unidades= 70g  (unidad) | c139 |  | elaboración propia a partir de receta |
| Pata Asada (Lonchas=25g) | c140 | 590 |  |
| Guisantes guisados GRAMOS | c141 |  |  |
| Ensaladilla Rusa (una ración = 120 gramos) | c142 |  | elaboración propia a partir de receta |
| Lentejas COMPUESTAS cucharón=100g | c143 |  | receta canaria: calculo a partir de sus ingrediente. |
| Bollería industrial gramos ) | c144 |  | ensaimadas=37g, valencianas=30g, tartaleta de manzana=74g, sobaos=20g, napolitanas=40g |
| Palmera (clásica, chocolate, integral) la grande 115 gramos, la pequeña 23 gramos) | c145 |  | composición envoltorio, palmera eidetesa |
| Tamarillo piezas =30g | c146 |  |  |
| Nocilla gramos | c147 |  | COMPOSICIÓN ENVOLTORIO NOCILLA |
| Palomitas de maíz (25g=ración) | c148 |  | composición envoltorio, palomitas de maíz Auchan |
| zumos envasados edulcorados ml | c149 |  | composición envoltorio, zumo pera-piña JUVER y Libby´s |
| YOGURT DESNATADO EDULCORADO | c150 |  | composición envoltorio HACENDADO |
| YOGURT NATURAL(AZUCARADO, CON Y SIN FRUTAS) | c151 | 558 ,567, 578 | yogurth natural azucarado, con frutas sin frutas, DANONE |
| YOGURT NATURAL GRIEGO | c152 |  | composición envoltorio HACENDADO |
| YOGURT DE CABRA | c153 |  | composición envoltorio marca Romar, mercadona |
| YOGURT SOJA/SIN LACTOSA | c154 |  | composición envoltorio marca HACENDADO Y KAIKU, mercadona |
| Arepa asada (50 gramos) | c155 |  | elaboración propia a partir de receta |
| Ciruelas (piezas) = 60 gramos | c156 | 129 |  |
| TORTILLA FRANCESA: un huevo 55 gr, dos huevos 110 gr | c157 |  | elaboración propia a partir de receta |
| Melocotón, Albaricoque, durazno | c158 | 243,272 |  |
| CROQUETAS (UNIDADES) 35 g c/u | c159 | 861 |  |
| Copa de Chocolate / nata de DANONE UNIDAD=150gr | c160 |  | calculado a partir de sus ingredientes (elaboración propia |
| tortitas de trigo para burritos unidades=40g | c161 |  | USDA: 18970, Tortillas, ready-to-bake or -fry, flour, shelf stable |
| QUESO CREMA FILADELPHIA | c162 |  | USDA: 01017, Cheese, cream |
| NUGGETS POLLO unidades, 26 g c/u | c163 |  | Elaboración a partir de receta. |
| PALITOS DE CANGREJO, UNIDADES=18g | c164 |  | envoltorio |
| PURÉ DE PAPAS, gramos | c165 | 983 | MATAIX: puré de patatas Maggi. Para azúcares y almidón: proporción de las papas |
| PIÑA NATURAL Y EN SU JUGO, gramos | c166 | 329 |  |
| CHAMPIÑÓN, setas, gramos | c167 | 195 |  |
| JUDIAS GUISADAS, BLANCAS PINTAS, ETC gramos | c168 | 216 |  |
| ENSALADA DE COL, ZANAHORIA, REMOLACHA, gramos | c169 |  | elaboración propia a partir de receta |
| AREPA FRITA (UNIDAD) 90 gramos unidad | c170 |  | elaboración propia a partir de receta |
| MAIZ DULCE (GRAMOS) | c171 | 62 |  |
| VINAGRE (ML) | c172 |  | USDA (Mataix para las proporciones de azúcares y almidón) |
| CACHAPA (UNIDAD) | c173 |  | elaboración propia a partir de receta |
| EMPANADA (unidad) | c174 |  | elaboración propia a partir de receta |
| TEQUEÑO (UNIDAD) | c175 |  | elaboración propia a partir de receta |
| PREPARADO DE GELATINA (UNIDAD = 125 GRAMOS) | c176 |  | etiqueta-gelatina Royal. |
| PLATANO SANCOCHADO (UNIDAD) | c177 |  | elaboración propia a partir de receta |
| SUSPIRO O BOLITA DE COCO (una bola pesa 20 gramos) | c178 |  | BOLA DE COCO DE MERCADONA: ETIQUETA. |
| HALLACA (UNIDAD) | c179 |  | elaboración propia a partir de receta |
| GUAYABA (PIEZAS) | c180 | 258 |  |
| higo pico o chumbo (unidades) | c181 | 260 |  |
| BERENJENA (HERVIDA) GRAMOS | c182 | 185 |  |
| DÁTIL (UNIDADES) UNA UNIDAD PESA 10 GRAMOS. | c183 | 252 |  |
| PUCHERO CANARIO (RACION DE 300 GR O 2 CUCHARONES) | c184 |  | Elaboración propia a patir de RECETA TRADICIONAL CANARIA |
| PASTEL DE MANZANA TEISOL (UNIDAD = 65 GR) | c185 |  | internet-pagina teisol- tipo azúcares aproximación según azúcar de manzana , agms, agpi aproximación. |
| GUSANITOS (RISI: BOLSA PESA 35 GRAMOS) | c186 |  | etiqueta: para cálculo de azúcares se asume que se trata de maiz cocido-Mataix. |
| SAN JACOBO O ROLLITO JAMON Y QUESO EMPANADO (pieza con aceite 102 gr) | c187 |  | Universidad de Murcia. Para San jacobo Findus. |
| POLVORON (40 GRAMOS) | c188 |  | elaboración propia a partir de receta |
| MERLUZA REBOSADA (GRAMOS). UNA VARITA DE MERLUZA 30 GRAMOS | c189 |  | PESCANOVA_ MÁS ACEITE. |
| CARNE EN SALSA (CUCHARON). CUCHARON = 150 GRAMOS, RACION = 270 GRAMOS. | c190 |  | elaboración propia a partir de receta |
| ARROZ 3 DELICIA (GRAMOS) | c191 |  | ELABOración propia a partir de receta |
| CALAMARES A LA ROMANA (UNA RACIÓN 120 GR) | c192 |  | Elaboración propia a partir de receta |
| CHURROS DE PESCADO (UN CHURRO PESA 25 GR) | c193 |  | elaboración propia a partir de receta |
| CHURROS O PORRAS (ración o unidad = 25 gr) | c194 |  | elaboración propia a partir de receta |
| ROLLITO DE PRIMAVERA (UNO PESA 89 GRAMOS) | c195 |  | USDA |
| CAQUI (gramos) | c196 | 245 |  |
| ACTIMEL LIQUIDO (UN BOTE 100 ML) | c197 | 359 | Mataix (ver ficha para azúcares) |
| MERENGUE (Un merengue =8 gramos) | c198 |  | National Institute of Finlandia (a partir de receta) |
| ALTRAMUCES (gramos) | c199 |  | USDA: 16077, Lupins, mature seeds, cooked, boiled, Azúcares = 0 (a partir de etiqueta de producto de mercadona; no se encuentra en otras fuentes) |
| NÍSPERO (unidades: peso aproximado 80 gr/unidad) | c200 | 278 | Mataix (azúcares proporción de los mostrados en dietas.net: glucosa 26,6 %; fructosa 37,3 % y sacarosa 36,1 %) |
| REMOLACHA O BETERRADA (gramos) | c201 | 230 |  |
| MORCILLA (gramos) | c202 | 655 | Mataix (los HC = 3gr, asumimos son todos sacarosa en base a USDA: 07005, Blood sausage) |
| LECITINA DE SOJA (gramos) | c203 |  | Marca Delyplus de Mercadona (azúcares=sacarosa) |
| ALMOJADABA (unidades o raciones: una=75 gr) | c204 |  | Elaboración propia a partir de receta |
| GAZPACHO (ml) | c205 |  | Elaboración porpia a partir de receta |
| ALMOGROTE (ml) | c206 |  | Elaboración porpia a partir de receta |
| CASTAÑA (unidad; una castaña = 10 gr) | c207 | 166 |  |
| PATÉ O FOAGRAS (gramos) | c208 | 658 | Mataix (cálculo de azúcares a partir de los porcentajes de la tabla Finlandesa) |
| TURRÓN DURO DE ALMENDRA (trozo=40 gr) | c209 |  | Elaboración a partir de receta. |
| COMPOTA DE MANZANA (gramos) | c210 |  | Elaboración porpia a partir de receta |
| PARCHITA-MARACUYA (gramos) | c211 |  | Danish database: nº 1055. |
| FRUCTOSA (azúcar ) | c212 |  |  |
| HAMBURGUESA DE SOJA | c213 |  | USDA 16147 |
| PELADILLAS (UNIDADES: UNA = 4 GRAMOS) | c214 |  | Elaboración porpia a partir de receta |
| GEL ENERGÉTICO | c215 |  | Ultra Gel 700 (Decathlon Brand) |
| GUISANTES COMPUESTOS | c216 |  | Homemade, traditional recipe |
| MORINGA: hoja seca (gram). | c217 |  | USDA |
| SESAMO, SEMILLAS (gram) | c218 |  | USDA |
| LASAÑA DE ATÚN (gram) | c219 |  | A partir de receta tradicional |
| LASAÑA DE CARNE (gram) | c220 |  | A partir de receta tradicional |
| LASAÑA DE VERDURA (gram) | c221 |  | A partir de receta tradicional |
| TRUCHA DE BATATA (unidad) | c222 |  | A partir de receta tradicional |
| TARTA DE CUALQUIER TIPO EXCEPTO DE MANZANA (gram) | c223 |  | A partir de receta tradicional |
| TARTA DE MANZANA (gram) | c224 |  | A partir de receta tradicional |
| CANELONES DE ESPINACA (gram) | c225 |  | A partir de receta tradicional |
| TIRAMISU (gram) | c226 |  | A partir de receta tradicional |
| HOLALDRE (gr) | c227 |  | A partir de receta tradicional |
| CABELLO DE ANGEL (gr) | c228 |  | A partir de receta tradicional |
| TRUCHA DE CALABAZA (Unidad = 100 gr) | c229 |  | A partir de receta tradicional |
| PAN DE LECHE (unidad = 40 gr)) | c230 |  | A partir de receta tradicional |
| ROSQUETE CANARIO (unidad = 50 gr) | c231 |  | A partir de receta tradicional |
| DULCE O TARTA DE MERENGUE ((gr) | c232 |  | A partir de receta tradicional |

Fuentes del contenido nutricional:

- Mataix (referencia 24)

- Base de datos española de composición de alimentos (BEDCA): <http://bedca.net/>

- United States Department of Agriculture. Agricultural research service. (USDA): <https://ndb.nal.usda.gov>

- National Institute for Health and Welfare, Fineli (Finnish Institute): <https://fineli.fi/fineli/en/index>?

- Danish food composition data: <http://frida.fooddata.dk/index.php?lang=en>

Comentarios acerca de los nombres de las especies de pescado en este anexo:

Los nombres comunes en español se obtuvieron de los pescados de las Islas Canarias y fueron verificados a partir de las guías oficiales de especies de interés comercial: (<http://pescamax.foroactivo.com/t720-peces-de-interes-pesquero-en-las-islas-canarias-por-www-jornadasdepesca-blogspot-com> ),
