# Supplementary material for: Effectiveness of a low-fructose and/or low-sucrose diet in decreasing insulin resistance (DISFRUTE study): study protocol for a randomized controlled trial
Source: Trials. 2017 Aug 7;18:369. doi: 10.1186/s13063-017-2043-z (PMC5547514; doi:10.1186/s13063-017-2043-z)
Supplement: Supplementary file 7 — Foods included to date and sources of macronutrients and sugars (English). (DOCX 40 kb) [file 13063_2017_2043_MOESM7_ESM.docx]

| FOOD | Ref. code | **Mataix reference** | **Name in food database or average value NOTES** |
| --- | --- | --- | --- |
| NON-WHOLE WHEAT BREAD (SANDWICH LOAF) (slice = 20 g) | c1 | 71 | BIMBO DOS brand sandwich loaf |
| WHOLE WHEAT BREAD (SANDWICH LOAF) (slice = 20 g ) | c2 | 74 |  |
| NON-WHOLE WHEAT BAGUETTE (grams) | c3 | 68 | White bread |
| WHOLE WHEAT BAGUETTE (grams) | c4 | 78 | Whole wheat bread |
| GOFIO (tablespoon = 20 g) | c5 | 46, 47 | Average: wheat-corn |
| CEREALS WITH ADDED SUGAR (large spoonful = 30 g) | c6 | 30, 27, 31, 32, 63, 87, 89 | Average: corn flakes with sugar (Kellogs), corn flakes with sugar (Pascual), puffed rice with honey (Pascual), toasted corn with honey and peanuts (Kellogs), puffed corn with honey, puffed wheat with honey, puffed wheat with sugar |
| CEREALS WITHOUT ADDED SUGAR (large spoonful = 30 g) | c7 | 13, 14, 28, 29, 33 | Average: toasted puffed rice (Pascual), toasted puffed rice (Kellosgs), toasted corn flakes (Kellogs), toasted corn (Pascual), rice and wheat flakes (Special K) |
| Fried potatoes (grams) | c8 |  | 30 mL olive oil per 100 g. Homemade |
| Potatoes in stew (serving = 60 g) | c9 | 221 | Boiled potato, per edible portion |
| TOAST (WHITE AND WHOLE WHEAT BREAD ) (slice = 11 g) | c10 |  | Ingredients from label (Hacendado brand) |
| PORK, SPARE RIBS, EXCLUDING PRESERVED MEATS IN CASING | c11 | 582, 584, 586, 594 | Chop, ribs, loin, tenderloin |
| BEEF, VEAL g | c12 | 605, 614, 618 | Lean beef, beef tenderloin |
| RABBIT, HARE g | c13 | 624 | Rabbit |
| CHICKEN, POULTRY g | c14 | 629, 630, 631, 636, 637 | Chicken, duck, turkey dark meat, chicken dark meat, chicken breast |
| CURED SERRANO HAM (slice = 30 g), (half a thin slice) | c15 | 650 |  |
| COOKED HAM, LOW-MOISTURE HIGH PROTEIN and REGULAR (slice= 20 g) | c16 | 649 | Cooked ham |
| TURKEY BREAST (slice = 20 g) | c17 | 661 | Turkey breast |
| FRESH LIVER (ORGAN MEAT) (grams) (similar to meatballs) | c18 | 585, 599, 609, 616, 635 | Pork, lamb, beef, chicken liver |
| LAMB (grams) | c19 | 596, 601 | Lamb chops and leg |
| EGGS (1 = 50 g) | c20 | 756 | Chicken eggs |
| HAMBURGERS (WITH BUN), 1 = 185 g | c21 | 80, 1028, 1021, 1015 | 60 g bun, 10 g mustard, 15 g ketchup |
| HOT DOGS (WITH BUN), 1 = 120 g | c22 | 664, 80, 1021, 1015 | 45 g bun, 50 g hot dog, 10 g mustard, 15 g ketchup |
| WHITE FISH (COMMON DENTEX, PARROTFISH, SPLENDID ALFONSINO, BLACKSPOT SEABREAM, WRECKFISH) (grams) | c23 | 668,; 687, 689, 693, 696, 697, 711, 714 | Inland grouper, gilt-head seabream, four-spot megrim, common sole, hake, grouper |
| BLUE FISH (TUNA, SARDINE, MACKEREL, RUBBERLIP GRUNT, MOROCCAN WHITE SEABREAM, BLACK SEABREAM) (grams) 5 | c24 | 673, 678, 692, 710, 681 | Tuna (albacore, skipjack tuna), chub mackerel, white trevally, horse mackerel, sardine |
| OCTOPUS, CUTTLEFISH, SQUID (grams) | c25 | 716, 727 | Squid, octopus |
| SHELLFISH (LIMPET, SHRIMP, CRAB) (grams) | c26 | 715, 717, 719, 721, 722, 724 | Clams (grooved carpet clam, striped venus clam), cockels, crab, velvet crab, spider crab, shrimp and popcorn shrimp, lobster and spiny lobster, mussels |
| SALTED OR DRIED FISH (grams) | c27 | 732, 733, 737, 738, 748 | Smoked herring, salted herring, smoked cod, salted cod, smoked salmon |
| FRESH SAUSAGE (grams) | c28 | 663, 664, 665 | Fresh sausage, frankfurter, Vienna sausage |
| ORGAN MEATS (TRIPE, KIDNEYS) (grams) | c29 | 583, 591, 597, 602, 603, 604, 607, 612, 613 | Pork, lamb and beef tripe, kidneys, heart, brains |
| PORK BELLY, BACON, CHITTERLINGS (slice = 18 g) | c30 | 595, 638, 642 | Pork belly, bacon, chitterlings |
| SPREADABLE CANARY ISLANDS SAUSAGE (1 = 40 g) | c31 | 667 | Sobrasada (soft cured sausage) |
| CURED MEATS IN CASINGS (slice = 20 g) | c32 | 643, 644, 645, 648, 653, 656, 662 | Pork bologna, turkey bologna, sausage, thin sausage (fuet), cured pork tenderloin, mortadella, salami |
| SATURN PEACH (small = 65 g, medium = 100 g, large = 135 g) | c33 |  | BEDCA database: for sugars, equivalent values for portions of peach (sacarose 7 g, fructose 1.6 g, glucose 1.6 g) |
| SALAD (grams) | c34 | 194, 217, 222, 226, 232, 236, 238, 239, 242, 768, 839 | Ingredients: onion, lettuce, cucumber, pepper, raw tomato, carrot, black olives, avocado, olive oil, vinegar |
| APPLE (grams) | c35 | 269, 270, 271 | Red, Golden, Granny Smith apple |
| PEAR (grams) | c36 | 280 | Pear |
| BANANA (grams) | c37 | 282 | Banana |
| ORANGE, TANGERINE, GRAPEFRUIT, LEMON (grams) | c38 | 265, 267, 276, 283 | Lemon, tangerine, grapefruit, orange |
| PAPAYA (grams SIMILAR TO SLICE OF MELON) | c39 | 279 | Papaya |
| GRAPES (1 HANDFUL OR 1 SMALL BOWL) (EQUIVALENT TO 200 grams) (small grapes = 6 g), (large grapes = 12 g) | c40 | 289, 290 | White and dark grapes |
| FRESH JUICE (fresh, not packaged) (mL) | c41 | 299, 307 | Fresh-squeezed lemon and orange juice |
| FRUIT JUICE (PACKAGED) (mL) | c42 | 296, 302, 304, 305 | Multifruit juice (Kasfruit), apple (Kasfruit), peach and grape (Kasfruit), orange (commercial brand) |
| PRESERVED FRUIT (SYRUP) (grams) | c43 | 314, 320, 329 | Fruit cocktail in syrup, peaches in syrup, pineapple in syrup |
| FRESH TOMATO (1 = 145 g) | c44 | 232 | Tomato |
| AVOCADO (1 = approx 150 g) | c45 | 242 | Avocado |
| OLIVES (1) | c46 | 238, 239 | Black and green olives |
| RAW ONION (1 = approx 110 g) | c47 | 194 | Raw onion |
| RAW PEPPER (RED OR GREEN) (1 = approx 120 g) | c48 | 226 | Raw pepper |
| CUCUMBER (1 = approx 150 g) | c49 | 222 | Cucumber |
| MANGO (1 = approx 230 g) | c50 | 268 | Mango |
| KIWI (1 = approx 90 g) | c51 | 262 | Kiwi |
| FRESH FIG (1 = approx 50 g) | c52a | 259 | Green and black figs |
| PRICKLY PEAR (1 = approx 60 g) | c52 | 260 | Prickly pear |
| LETTUCE (grams) | c53 | 217 | Lettuce |
| RAW OR PRESERVED GARLIC (CLOVE) | c54 | 179 | Garlic |
| POWDERED MILD (DRINK) mL | c55 | 455 | Powdered milk |
| GOAT MILK mL | c56 | 428 | Goat milk |
| LIQUID WHOLE MILK mL | c57 | 445 | Cow milk (whole) |
| LIQUID LOW-FAT MILK mL | c58 | 439 | UHT low-fat milk |
| LIQUID HALF-FAT MILK mL (and DANACOL brand = 100 mL) | c59 | 461 | UHT half-fat milk (including Danacol) |
| MILK WITH VEGETABLE FAT (DAIRY PRODUCTS) mL | c60 | 435, 438 | Low-fat mild with vegetable fat (olive oil) (Lilac), low-fat milk with vegetable oils |
| CONDENSED MILK (teaspoon = 10 g) | c61 | 430 | Condensed milk |
| SOY MILK (mL) | c62 |  | Sugar content from label (Hacendado brand). Others Mataix reference |
| CHOCOLATE SOY MILK (mL) | c63 |  | Sugar content from label (Hacendado brand) |
| PETIT SUISSE (MANUFACTURED) (serving = 60 g) | c64 | 486, 489, 490, 493 | Low-fat petit suisse with fruit (Danone brand), with fruit and cereals (Danone), plain with sugar (Danone), other flavors (Danone) |
| FLAN, VANILLA PUDDING (MANUFACTURED) (SERVING = 140 g) | c65 | 410, 413, 476, 479 | Egg flan (Danone brand); vanilla flan (Danone), chocolate pudding (Danone), vanilla pudding (Nestle brand) |
| FLAVORED MILKSHAKE (MANUFACTURED) (mL) | c66 | 367, 369, 373, 375 | Cocoa milkshake (Puleva); low calorie chocolate (McDonalds), low-calorie vanilla (McDonalds), vanilla UHT (Puleva) |
| DIARY CREAM (mL) | c67 | 470, 473, 474 | Cream, pasteurized cream (Puleva brand), UHT cream (Puleva) |
| FRESH WHITE (UNCURED) CHEESE (g) | c68 | 495, 506 | White low-fat cheese, fresh low-fat cheese (Danone brand), Burgos cheese (Arias brand) |
| SEMICURED CHEESE (YELLOW OR WHITE) (g) | c69 | 519, 523 | Semicured manchego cheese, semicured full-fat cheese (Puleva brand) |
| CURED CHEESE (YELLOW OR WHITE) (g) | c70 | 505, 517, 520 | Emmenthal, cured manchego, Parmesan |
| BUTTER (teaspoon = 20 g) | c71 | 776 | Salt-free butter |
| MARGARINE (teaspoon = 20 g) | c72 | 780 | Margarine |
| OLIVE OIL (teaspoon = 5 g) (tablespoon = 10 g) | c73 | 768 | Olive oil |
| OTHER VEGETABLE OILS (teaspoon = 5 g) | c73bis | 762, 765, 767, 771 | Peanut, sunflower, corn, soy |
| MAYONNAISE (teaspoon = 10 g) | c74 | 783 | Commercial mayonnaise |
| KETCHUP AND OTHER PROCESSED TOMATO SAUCES (teaspoon = 10 g) | c75 | 1015, 1026, 1027 | Ketchup, uncooked strained tomato (Solis brand), fried tomato sauce (Orlando brand) |
| DRIED NUTS (PEANUTS, ALMONDS, WALNUTS) (tablespoon = 60 g or 1 handful) | c76 | 348, 351, 352, 353, 356 | Almonds, hazelnuts, peanuts, chestnuts, walnuts |
| MOJO PICON SAUCE, TABASCO SAUCE (teaspoon = 15 g) | c77 | 179, 196 225, 768, 839 | Garlic, red chilli, black pepper, olive oil, vinegar |
| MUSTARD (teaspoon = 10 g) | c78 | 1016 | Mustard |
| SUGAR (g) | c79 | 131 | Sugar |
| HONEY (teaspoon = 10 g) | c80 | 167 | Honey |
| ICE CREAM (serving = 80 g) | c81 | 418 | Ice cream, Mataix (proportion of carbohydrates: Finnish Institute: food no. 663: 69.3% sacarose and 30.7% lactose) |
| POPSICLE (1 =70 g) or SHERBET | c82 |  | Finnish Institute: Food no. 31991 Sherbet (Carte D’or brand) |
| PLAIN WHOLE WHEAT FLOUR /MARÍA Brand COOKIES (1 = 6 g) | c83 |  | Ingredients from label (Hacendado brand) |
| TRADITIONAL YELLOW CAKE (1 slice) | c84 | 55, 131, 444 , 776, 837 | Wheat flour, sugar, whole milk, eggs, butter, leavening agent. Homemade |
| UNFILLED COOKIES CREMA (MARÍA brand) Number of cookies | c85 | 118 | María brand cookies |
| FILLED COOKIES, including chocolate filling | c86 | 113, 117, 120 | Chocolate cookies, Chocolate digestive biscuits, cocoa cream-filled ladyfingers (Dulcesol brand) |
| FILTERED COFFEE (DRIP COFFEE) (small cup) | c87 |  | Expresso coffee |
| EXPRESSO COFFEE (home or cafés/restaurants) (mL) | c88 | 796 |  |
| DECAFFINATED COFFEE (mL) | c89 | 796 |  |
| INSTANT POWDERED CHOCOLATE DRINK (CONTAINING ADDED SUGAR (ColaCao, teaspoon = 5 g) | c90 | 793, 792 | Powdered cocoa with sugar, ingredients from label (ColaCao brand), soluble cocoa (Nesquik brand) |
| CHOCOLATE (BARS, SQUARES, INDIVIDUAL CHOCOLATES) (small bar = 40 g) | c91 | 135, 136, 141, 144, 161, 162 | Individual chocolates (Nestle brand), dark chocolate, white chocolate, extra fine milk chocolate with almonds (Nestle), extra fine dark chocolate with almonds (Nestle) |
| QUINCE OR GUAYABA QUINCE (slice = 15 g) | c92 | 321 | Quince and fruit pastes |
| MARMALADE (spoonful = 12 g) | c93 | 322, 324,3 27 | Apricot, plum, orange marmalade |
| HARD CANDIES, OTHER CANDIES (1 = 5 g) | c94 | 138, 14 | Soft candies and chewing gum with sugar, including pacifier-shaped and round |
| DRIED FIGS (1 = 17 g) | c95 | 333 | Dried figs |
| RAISINS (large spoonful) | c95bis | 335 | Raisins |
| WATERMELON (slice) | c96 | 285 | Watermelon |
| MELON (slice) | c97 | 273 | Melon |
| STRAWBERRIES (1) | c98 | 254 | Strawberries |
| Cherries (1) | c99 | 246 | Cherries |
| WATERCRESS (large spoonful) | c100 | 186 | Watercress |
| HOT PEPPERS (WHOLE, NOT FOR MOJO SAUCE) (1) | c101 | 196, 197 | Red chilli, green chilli |
| RICE (prepared in any manner) (grams) | c102 | 8, 9, 10, 11 | Uncooked brown rice, boiled brown rice, uncooked milled rice, boiled milled rice |
| COOKED VEGETABLE (VEGETABLE BEAN, CARROT, ZUCCHINI SQUASH, CABBAGE, ROUND ZUCCHINI, PUMPKIN, COLIFLOWER) (grams) | c103 | 190, 192, 204, 206, 216, 237 | Boiled zucchini squash, vegetable beans, pumpkin, carrots, cabbage, coliflower |
| CORN CAKES (1) | c104 |  | Ingredients from label (Hacendado brand). Sugar assumed to be sacarose. Fat assumed to be 50% mono- and 50% polyunsaturated, saturated fats (according to label) subtracted from total fats |
| WHOLE GRAIN CEREALS (large spoonful = 30 g) | c105 | 22,66 | All Bran (Kellogs brand), wheat bran (Hacendado brand), muesli (Kellogs) |
| CHOCOLATE-FILLED CEREALS (large spoonful = 30 g) | c106 |  | Chocolate-filled cereal (Hacendado brand) |
| TEA (mL) | c107 | 826 | Tea |
| SOFT DRINKS (LIGHT) (mL) | c108 | 788 | Carbonated soft drinks (light) |
| SOFT DRINKS (REGULAR), CARBONATED (mL) | c109 | 822 | Cola flavored soft drink |
| WINE, CAVA , CHAMPAGNE (mL) | c110 | 804, 833 | Cava, table wine |
| BEER, CIDER (mL) | c111 | 805, 806,8 24 | Beer, dark beer, cider |
| LIQUEURS AND SWEET WINES (mL) | c112 | 820, 821, 830, 831 | Coffee liqueur, other liqueurs, sweet wine (Malaga), muscatel wine (glassful) |
| RUM AND COGNAC (mL) | c113 | 814, 823 | Rum, cognac |
| BRANDY, WHISKY (mL) | c114 | 786, 835 | Brandy, whisky |
| TAP WATER (mL) | c115 |  |  |
| BOTTLED WATER, CARBONATED (mL) | c116 | 785, 825 | Mineral water (Perrier), soda water |
| BOTTLED WATER, STILL (mL) | c117 | 784 | Mineral water |
| SOFT DRINKS, NON-CARBONATED (mL) | c117bis | 789, 790 | Isotonic drinks (Aquarius, Gatorade) |
| SALT (spoonful) | c118 |  | Low-salt diet at [www.fisterra.com](http://www.fisterra.com). Dr María Carreira Miño |
| CANNED SARDINES OR TUNA (can = 85 g) | c119 | 734, 735, 749 | Tuna in oil, tuna in brine, sardines in oil |
| ÑAME, SWEET POTATO (grams) | c120 | 183 | Sweet potato |
| HERBAL TEA 1 (mL) | c121 | 826 |  |
| DRIED INSTANT SOUP (large spoonful = 100 g) | c122 | 989, 992, 994, 998, 999 | Poultry with rice, chicken noodle, beef with vegetables, with small pasta (maravilla type) |
| PASTA COOKED AT HOME (MACARONI, SPAGHETTI, etc.) (grams) p. 20-21 | c123 | 38, 41, 42, 59, 61 | Whole wheat boiled spaghetti, regular boiled spaghetti with salt, regular boiled spaghetti without salt, boiled lasagna, boiled macaroni |
| STEW (large spoonful) | c124 | 62, 176, 179, 191, 194, 189, 215, 220, 223, 232, 236, 608, 768, 992 | Corn, chard, garlic, pumpkin, onion, zucchini squash, green beans, potato, parsley, fresh tomato, carrot, beef ribs, olive oil |
| CANNED FOODS (STEWS, ETC.) (large spoonful) | c125 | 921, 923, 934, 937 | Tripe with tomato and pepper (Tila brand), traditional chickpea stew (Litoral brand), Asturian white bean stew (fabada) (Litoral), lentils with sausage (Litoral) |
| PRECOOKED PIZZA (grams) 1 whole = 350 g, 1 slice = 44 g | c126 | 895, 899, 903 | Tuna pizza, ham and cheese pizza, plain pizza (tomato sauce and cheese) |
| RANCHO CANARIO (large spoonful = 100 g | c127 |  | Homemade, traditional recipe |
| TUNA AND VEGETABLE SALAD | c128 |  | Homemade, traditional recipe |
| POTATO CHIPS (p. 92) | c129 | 1014 |  |
| CROISSANT (1 = 100 g) | c130 | 104 |  |
| CHOCOLATE-FILLED CROISSANT (1 = 100 g) | c131 | 105 |  |
| PLAIN MUFFIN (grams) | c132 | 119 |  |
| DONUT (PLAIN OR CHOCOLATE-COVERED (1 = 65 g) | c133 | 107, 108 | Average: Plain and chocolate-covered donuts |
| Honey bee pollen (teaspoon = 10 g) | c134 |  | Ingredients from label honey bee pollen (Hacendado brand) (sugar: apiculture journal 1993; no. 59 May) |
| VEGETABLE BEAN SALAD, grams | c135 |  | Homemade, traditional recipe |
| Escaldón de gofio (porridge with stock and toasted flour) (serving = 150 g) | c136 |  | Traditional recipe; content based on ingredients (1 serving = 150 g) not including mojo sauce |
| Potato omelette (grams) | c137 |  | Homemade, traditional recipe |
| Chickpea stew (large spoonful = 100 g) | c138 |  | Homemade, traditional recipe; also includes traditional dish from leftover stew (ropa vieja) |
| FRIED EGG (1 = 70 g)  (one) | c139 |  | Homemade, traditional recipe |
| Roast leg of pork (slice = 25 g) | c140 | 590 |  |
| Cooked peas (grams) | c141 |  |  |
| Potato salad (serving = 120 g) | c142 |  | Homemade, traditional recipe |
| Lentil stew (large spoonful = 100 g) | c143 |  | Traditional recipe; content based on ingredients |
| Sweet buns, rolls, muffins, etc. (grams) | c144 |  | Ensaimada = 37 g, valenciana = 30 g, apple tart = 74 g, sobao = 20 g, ladyfingers = 40 g |
| Flaky scroll pastry (plain, chocolate-covered, whole wheat) large = 115 g, small = 23 g) | c145 |  | Ingredients from label (Eidetesa brand) |
| Tree tomato (1 = 30 g) | c146 |  |  |
| Hazelnut and chocolate spread (grams) | c147 |  | Ingredients from label (Nocilla brand) |
| Popcorn (serving = 25 g) | c148 |  | Ingredients from label (Auchan brand popcorn) |
| Sweetened fruit juice (mL) | c149 |  | Ingredients from label, pear-pineapple juice (Juver and Libby´s brands) |
| LOW-FAT SWEETENED YOGURT | c150 |  | Ingredients from label (Hacendado brand) |
| PLAIN YOGURT (SWEETENED, WITH OR WITHOUT FRUIT) | c151 | 558, 567, 578 | Plain yogurt with sugar, with or without fruit (Danone brand) |
| PLAIN GREEK YOGURT | c152 |  | Ingredients from label (Hacendado brand) |
| GOAT MILK YOGURT | c153 |  | Ingredients from label (Romar brand, Mercadona) |
| SOY/LACTOSE-FREE YOGURT | c154 |  | Ingredients from label (Hacendado and Kaiku brands, Mercadona) |
| Baked flat bread (arepa) (50 grams) | c155 |  | Homemade, traditional recipe |
| Plums (1 = 60 g) | c156 | 129 |  |
| PLAIN OMELETTE: 1 egg 55 g, 2 eggs 110 g | c157 |  | Homemade, traditional recipe |
| Peach, Apricot | c158 | 243, 272 |  |
| CROQUETTES (1 = 35 g) | c159 | 861 |  |
| Copa de Chocolate/nata de DANONE (1 = 150 g) | c160 |  | Calculated from ingredients (Homemade) |
| Wheat flatbread for burritos (1 = 40 g) | c161 |  | USDA: 18970, Tortillas, ready-to-bake or -fry, flour, shelf-stable |
| CREAM CHEESE (PHILADELPHIA brand) | c162 |  | USDA: 01017, Cheese, cream |
| CHICKEN NUGGETS (1 = 26 g) | c163 |  | Homemade, traditional recipe |
| SURIMI (1 = 18 g) | c164 |  | Package |
| POTATO PURÉE, grams | c165 | 983 | Mataix: Potato purée (Maggi brand). Sugar and starch content estimated from proportions in potato |
| FRESH PINEAPPLE WITH JUICE, grams | c166 | 329 |  |
| COMMON MUSHROOMS, OYSTER MUSHROOMS, grams | c167 | 195 |  |
| LEGUME BEANS, grams | c168 | 216 |  |
| COLESLAW, CARROT SALAD, BEET SALAD, grams | c169 |  | Homemade, traditional recipe |
| FRIED FLAT BREAD (AREPA) (1 = 90 g) | c170 |  | Homemade, traditional recipe |
| SWEET CORN (grams) | c171 | 62 |  |
| VINEGAR (mL) | c172 |  | USDA (Mataix table for proportions of sugars and starch) |
| CACHAPA (thick crepe) (1) | c173 |  | Homemade, traditional recipe |
| SMALL MEAT PIE (1) | c174 |  | Homemade, traditional recipe |
| TEQUEÑO (fried bread stick) (1) | c175 |  | Homemade, traditional recipe |
| INSTANT GELATIN (1 = 125 g) | c176 |  | Ingredients from label (Royal brand) |
| SANCOCHADO (meat or fish, potato, vegetables, porridge) BANANA (1) | c177 |  | Homemade, traditional recipe |
| COCONUT PASTRY (1 = 20 g) | c178 |  | MERCADONA Brand, Ingredients from label |
| HALLACA (tamale) (1) | c179 |  | Homemade, traditional recipe |
| GUAYABA (1) | c180 | 258 |  |
| Prickly pear (1) | c181 | 260 |  |
| EGGPLANT (AUBERGINE) (COOKED) grams | c182 | 185 |  |
| DATES (1 = 10 g) | c183 | 252 |  |
| PUCHERO CANARIO (Canary Islands stew: chickpeas, vegetables, meats, poultry) (serving = 300 g) | c184 |  | Homemade, traditional recipe |
| Apple pastry (manufactured commercial brand) (1 = 65 g) | c185 |  | Teisol internet site. Approximate sugar content based on apple, monosaturated and polyunsaturated fatty acids |
| CHEESE PUFFS (Risi brand, 1 package = 35 g) | c186 |  | Label: sugar content estimated assuming cooked corn (Mataix) |
| (SAN JACOBO) BREADED FRIED HAM AND CHEESE ROLL (1 with oil = 102 g) | c187 |  | University of Murcia. Findus brand San Jacobo |
| POLVORÓN (SHORTBREAD COOKIE) (40 grams) | c188 |  | Homemade, traditional recipe |
| FRIED BATTER-COATED HAKE (grams) 1 HAKE FISH STICK (1 = 30 g) | c189 |  | Pescanova brand, plus oil for frying |
| STEWED BEEF (large spoonful = 150 g, serving = 270 g) | c190 |  | Homemade, traditional recipe |
| FRIED RICE WITH EGG, VEGETABLE AND MEAT OR SHRIMP (grams) | c191 |  | Homemade, traditional recipe |
| FRIED BATTER-COATED SQUID (serving = 120 g) | c192 |  | Homemade, traditional recipe |
| FRIED BATTER-COATED FISH (1 = 25 g) | c193 |  | Homemade, traditional recipe |
| FRIED DOUGH (1 = 25 g) | c194 |  | Homemade, traditional recipe |
| SPRING ROLL (1 = 89 g) | c195 |  | USDA |
| PERSIMMON (grams) | c196 | 245 |  |
| LIQUID YOGURT (Actimel/DanActive probiotic) (1 = 100 mL) | c197 | 359 | Mataix (see entry for sugars) |
| MERINGUE (1 = 8 g) | c198 |  | Finnish Institute (homemade, traditional recipe) |
| LUPINES (grams) | c199 |  | USDA: 16077, Lupines, ripe seeds, cooked, boiled, sugar = 0 (Ingredients from label, Mercadona brand; no other sources found) |
| MEDLAR (1 = approx 80 g) | c200 | 278 | Mataix (sugar according to dietary proportion in www.dietas.net: glucose 26.6%; fructose 37.3%, sucrose 36.1%) |
| BEET (grams) | c201 | 230 |  |
| BLOOD SAUSAGE (grams) | c202 | 655 | Mataix (carbohydrates = 3 g, assuming all are sucrose according to USDA: 07005, blood sausage) |
| SOY LECITHIN (grams) | c203 |  | Delyplus brand (Mercadona) (sugar content = sucrose) |
| ALMOJÁBANA (cheese cake) (1 serving = 75 g) | c204 |  | Homemade, traditional recipe |
| GAZPACHO (mL) | c205 |  | Homemade, traditional recipe |
| ALMOGROTE (cheese and pepper spread) (mL) | c206 |  | Homemade, traditional recipe |
| CHESTNUTS (1 = 10 g) | c207 | 166 |  |
| PATÉ (FOIE GRAS OR OTHER) (grams) | c208 | 658 | Mataix (sugar content calculated from percentages in Finnish Institute table) |
| HARD ALMOND NOUGAT (1 piece = 40 g) | c209 |  | Homemade, traditional recipe |
| APPLE COMPOTE (grams) | c210 |  | Homemade, traditional recipe |
| PASSION FRUIT (grams) | c211 |  | Danish database: no. 1055 |
| FRUCTOSE (sugar ) | c212 |  |  |
| SOY HAMBURGER | c213 |  | USDA 16147 |
| SUGARED ALMONDS (1 = 4 g) | c214 |  | Homemade, traditional recipe |
| ENERGY GEL | c215 |  | Ultra Gel 700 (Decathlon Brand) |
| PEAS WITH OTHER VEGETABLES, SAUSAGE AND EGG | c216 |  | Homemade, traditional recipe |
| MORINGA (drumstick tree): dried leaves (grams) | c217 |  | USDA |
| SESAME SEEDS (grams) | c218 |  | USDA |
| TUNA LASAGNA (grams) | c219 |  | Homemade, traditional recipe |
| MEAT LASAGNA (grams) | c220 |  | Homemade, traditional recipe |
| VEGETABLE LASAGNA (grams) | c221 |  | Homemade, traditional recipe |
| TRUCHA DE BATATA (yam, almond and sugar-filled pastry) (1 portion) | c222 |  | Homemade, traditional recipe |
| CAKE OF ANY FLAVOR OR TYPE EXCEPT APPLE (grams) | c223 |  | Homemade, traditional recipe |
| APPLE CAKE (grams) | c224 |  | Homemade, traditional recipe |
| SPINACH-FILLED CANNELLONI (grams) | c225 |  | Homemade, traditional recipe |
| TIRAMISU (grams) | c226 |  | Homemade, traditional recipe |
| Flakey pastry (g) | c227 |  | Homemade, traditional recipe |
| Candied pumpkin or squash (g) | c228 |  | Homemade, traditional recipe |
| TRUCHA DE CALABAZA (candied, spiced pumpkin- or squash-filled pastry) (1 portion = 100 g) | c229 |  | Homemade, traditional recipe |
| PAN DE LECHE (sweet roll) (1 portion = 40 g) | c230 |  | Homemade, traditional recipe |
| Fried anisette-flavored donut (1 portion = 50 g) | c231 |  | Homemade, traditional recipe |
| Meringue-topped cake (g) | c232 |  | Homemade, traditional recipe |

Sources for nutrient contents:

- Mataix (reference 24)

- Base de datos española de composición de alimentos (BEDCA): <http://bedca.net/>

- United States Department of Agriculture. Agricultural research service. (USDA): <https://ndb.nal.usda.gov>

- National Institute for Health and Welfare, Fineli (Finnish Institute): <https://fineli.fi/fineli/en/index>?

- Danish food composition data: <http://frida.fooddata.dk/index.php?lang=en>

Note about the names of fish species in this Annex:

The common name in Spanish as used locally in the Canary Islands was verified against the regional fisheries authority's official guides to species of commercial interest (<http://pescamax.foroactivo.com/t720-peces-de-interes-pesquero-en-las-islas-canarias-por-www-jornadasdepesca-blogspot-com> ), and once the Latin name of the species was identified, the common name in English was obtained from Fishbase (<http://www.fishbase.org/search.php>).
